# On the Breakage of High Aspect Ratio Crystals in Filter Beds under Continuous Percolation

**DOI:** 10.1007/s11095-020-02958-x

**Published:** 2020-10-29

**Authors:** F. M. Mahdi, A. P. Shier, I. S. Fragkopoulos, J. Carr, P. Gajjar, F. L. Muller

**Affiliations:** 1 School of Chemical and Process Engineering, University of Leeds, Leeds, LS2 9JT UK; 2 Henry Moseley X-ray Imaging Facility, Henry Royce Institute for Advanced Materials, Department of Materials, The University of Manchester, Manchester, M13 9PL UK

**Keywords:** crystal breakage, percolation, pharmaceutical materials, pressure filtration, X-ray computed tomography (XCT)

## Abstract

**Purpose:**

This work details experimental observations on the effect of liquid flow percolating through packed beds of crystals to elucidate how the filtration pressure severely alters the size distribution and crystal shape. Pressure filtration is widely used in the pharmaceutical industry, and frequently results in undesired size distribution changes that hinder further processing.

**Methods:**

The percolation methodology presented fixes fluid flow through a bed of crystals, resulting in a pressure over the bed. X-ray computed tomography (XCT) provided detailed observations of the bed structure. Detailed 2D particle size data was obtained using automated microscopy and was analysed using an in-house developed tool.

**Results:**

Crystal breakage is observed when the applied pressure exceeds a critical pressure: 0.5–1 bar for ibuprofen, 1–2 bar for *β*-L glutamic acid (LGA) and 2–2.5 bar for para amino benzoic acid (PABA). X-ray computed tomography showed significant changes in bed density under the applied pressure. Size analysis and microscope observations showed two modes of breakage: (i) snapping of long crystals and (ii) shattering of crystals.

**Conclusion:**

LGA and PABA have a similar breakage strength (50 MPa), ibuprofen is significantly weaker (9 MPa). Available breakage strength data may be correlated to the volumetric Gibbs free energy. Data from 12 and 35 mm bed diameters compares well to literature data in a 80 mm filter; the smaller, easy to operate percolation unit is a versatile tool to assess crystal breakage in filtration operations.

**Electronic supplementary material:**

The online version of this article (10.1007/s11095-020-02958-x) contains supplementary material, which is available to authorized users.

## Introduction

Needle-shaped crystals with a high aspect ratio are common in pharmaceutical and fine chemicals processes (1,2) and their breakage during crystallisation has been widely investigated (3–5). When considering that particle/powder properties strongly influence the effectiveness of downstream operations, the desire to control these properties through particle engineering is a key facet of the modern crystallisation process (4). Particle engineering involves regulating the super saturation, hydrodynamic conditions and/or addition of seed to impart control over the particle size distribution (PSD), purity and polymorphic form of the resultant crystal product. However, downstream operations such as filtration, washing and drying relinquish some of the controlled benefits imparted by the crystallisation, thereby presenting an issue.

Isolation of crystals from the slurry is commonly carried out in a single agitated filter dryer (e.g. Nutsche filter dryer), or by centrifugation followed by drying (6,7). A crystal slurry is charged to the filter and pressurised, resulting in fluid flow through the filter cloth, leaving crystals behind as a permeable cake (6,8–11). The cake is then dried by application of either vacuum, or nitrogen flow. As moisture content nears the equilibrium the cake is agitated so as to ensure homogeneity and mobility of the dry solid produced. The PSD of the final product can, as industry observes, dramatically change post isolation (3,12,13).

Previous studies, in particular by Lekhal *et al*. (14,15), investigated the effect of temperature (20 to 80°C) and agitation speed (10 to 50 rpm) on the size and shape distribution of potassium chloride crystals. They assumed that the effect of particle breakage is mainly due to the use of agitated drying. Hamilton *et al*. (16) studied the breakage of needle-shaped cellobiose octaacetate. In this system continuous agitation during drying led to significant particle size changes. Applying the common practice of intermittent agitation minimised the size changes due to the significant reduction in the time particles were exposed to shear forces.

More recently, Macleod and Muller (3) investigated needle-shaped crystals of four different materials: acetylsalicylic acid (aspirin), acetaminophen (paracetamol), acetanilide and glutamic acid in laboratory and pilot scale filtration. They evaluate the PSD before and after pressure filtration at 0.5 to 4 barg and during drying. They found that the majority of needle-shaped particles breakage occurs during the filtration step, with further breakage occurring during agitation and drying.

Cornehl *et al*. (17,18) investigated the breakage of lysozyme crystals after cake filtration using two different habits (needle-shaped and isometric) at an applied pressure between 0.25 and 1 barg. The isometric crystals mostly suffered from attrition and chipping of edges from the larger particles, whilst the needles were completely broken/crushed. They noticed that crystal breakage occurs virtually instantaneously on application of pressure, whilst total failure/crushing occurred within 1 to 2 min of compression.

Breakage is generally divided into two categories: attrition and fragmentation. Attrition or erosion refers to the process by which fines are removed from the surfaces or edges of a parent particle, resulting in a gradual change in the parent size. Fragmentation refers to catastrophic breakage of a particle and results in a set of smaller particles. These two modes of breakage apply to primary as well as aggregate particles (5). As an object is subjected to load, energy is absorbed in the form of strain until a critical point is reached and the object yields (19). Typically, breakage occurs at points of structural instability where stresses concentrate, such as cracks, joints and weak bonds, with the extent of curvature governing the extent to which the stresses magnify. Forces in beds of particles are transferred via particle-particle contact points and, at constant overall system stress, fewer contact points increases the force loading per point and results in higher stresses within particles (3,20). The propensity for materials to fracture as a result of these forces is largely dependent on the particle shape and the intrinsic properties such as the tensile strength, toughness, Young’s Modulus, hardness, surface energy, moisture content and the material deformation behaviour (21).

In short, the impact of both filtration and drying on the PSD should be considered when developing an isolation process for a new development compound. In this paper, we address breakage during pressure filtration and present detailed experimental observations on the effect of liquid flow percolating through packed beds of three types of crystals *β*−L glutamic acid (LGA), 2-(4-isobutylphenyl) propanoic acid (Ibuprofen, racemic mixture) and *α*−para amino benzoic acid (PABA). From a material science point of view, these three materials have lattices that require breaking of very different inter molecular forces. Breakage across the needle axes involves breaking singular H-bonds (LGA, (22)), van der Waals forces (ibuprofen, (23)) and pi-pi interactions (PABA, (24)). Our premise is that the varying intermolecular forces result in significantly different breakage strength and behaviour on compression of the particles under liquid percolation.

Using a novel algorithm to compile accurate 2D particle size distributions from particle image data we observe breakage in packed beds to be a function of the applied percolation pressure over these crystals. Breakage is also confirmed using optical microscopy, scanning electron microscope (SEM) and X-ray computed tomography (XCT). From the observed extent of breakage, we estimate the critical breakage stress of the materials to correlate with the volumetric Gibbs free energy of the crystal lattices.

## Materials and Methodology

### Crystallisation Procedure

**Fig. 1:**
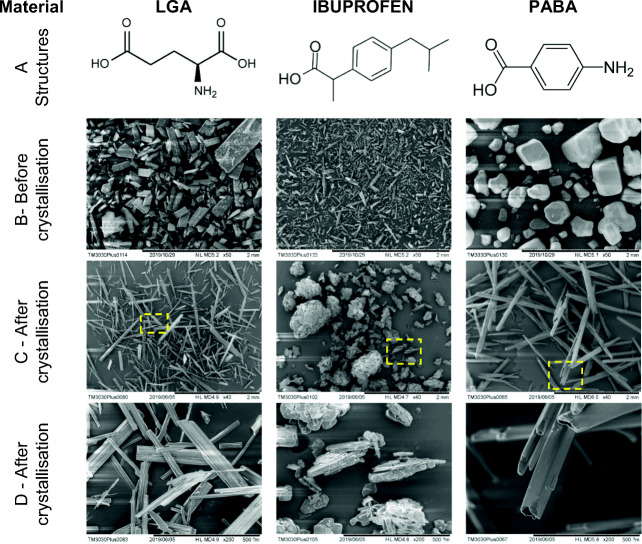
The three materials studied in this work: (**a**) the chemical structure; (**b**) SEM images of the raw material as received; (**c**, **d**) SEM images of the reference material at 20x (**c**) and 40x (**d**) magnification.

Three different organic materials were used in this work: (i) *β*-L-glutamic acid (99% purity, Sigma-Aldrich); (ii) 2-(4-isobutylphenyl) propanoic acid (racemic mixture, 98% purity, Tokyo Chemical Industry) and (iii) *α*-para-aminobenzoic acid (99% purity, Sigma-Aldrich). Figure 1 shows the SEM images of the initial materials before crystallisation and the reference materials (after crystallisation as explained in section 2.2.2).

A calorimeter (Simular calorimeter, H.E.L. group) consisting of a 1 L jacketed glass vessel fitted with a double pitched-blade Teflon agitator, a water-cooled condenser to catch any reflux and heater/chiller (FP32/HL, Julabo) temperature control was used to recrystallise materials. A Pt100 temperature probe, housed inside the glass vessel, was used to control the process. The crystallisation parameters were optimised for each material to produce the required crystal morphology and size. For all experiments, the crystallised slurries were held in the reactor at 20°C until required for percolation.

- *LGA* (β L-glutamic acid): 40 g LGA was charged to 1 L of deionised water and dissolved by holding at 70°C for 60 min with agitation at 300 rpm. The solution was cooled to 60°C to supersaturate the solution upon which seed material was added (3 wt% - 1.2 g, crushed LGA needles). The solution was held for 150 min at 60°C to allow the seed to grow, before cooling to 20°C over 12 h.
- *Ibuprofen* (Racemic, 2-(4-isobutylphenyl)propanoic acid): 60 g ibuprofen was charged to the vessel with 550 mL deionised water and 450 mL ethanol and dissolved by holding at 75°C for 60 min with 200 rpm agitation. The solution was cooled to 58°C to supersaturate the solution and seed material was added (3 wt% - 1.8 g, crushed Ibuprofen crystals). The solution was held for 120 min at 58°C and then cooled to 30°C in three steps at −0.06°C/min with a 120 min holding period following each step. Finally, the mixture was cooled from 30°C to 20°C over 7.5 h.
- *PABA* (α−para-aminobenzoic acid): 135 g PABA was charged to 700 mL deionised water and 300 mL ethanol and dissolved by holding at 70°C for 60 min with 300 rpm agitation. The solution was cooled to 50°C to supersaturate the solution and spontaneous nucleation. The solution was held for 60 min at 50°C, followed by cooling to 20°C over 12 h.

### Continuous Pressure Percolation

#### Percolation System

**Fig. 2:**
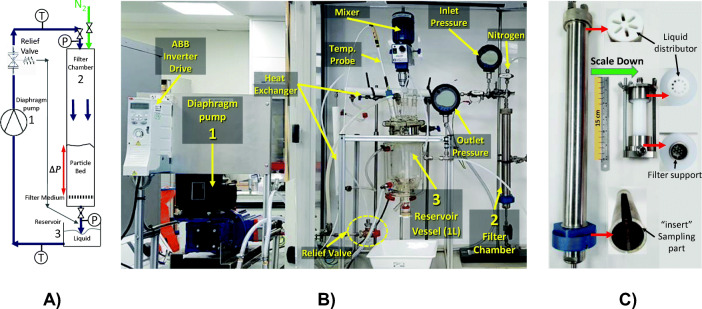
(**a**) Diagram of the experimental setup, (**b**) the continuous pressure percolation rig, and (**c**) details of the filter chambers at large and small scale.

The bench-scale continuous pressure percolation rig (Fig. 2a, b) recirculates the crystal slurries mother liquors from an atmospheric jacketed reservoir (1 L) using a high pressure diaphragm pump (Hydra-Cell P200 diaphragm pump Wanner, Engineering Inc.) equipped with metallic head and inverter frequency drive (IP20-UL) through a heat exchanger at 20°C and then into the filter assembly. The filter assembly has a distributor plate to break up the high-velocity jet of liquid on entry to the filter bed chamber. The filter support for the large filter assembly (ID-35) is a sintered porous stainless plate covered with filter paper (Whatman No 1), whilst the small filter assembly (ID-12) is a 440 μm element kit filter (Swagelok) covered with the same type of filter paper. Liquid passes through the particle cake and flows back into the recirculation vessel. The system was pressure-tested and protected from overpressure by a pressure regulator set at a maximum pressure of 7 bar. The fluid flow rate and empty system resistance were calibrated, see electronic supplementary information (ESI), section 1.

Control of the mother liquor temperature is important to avoid dissolution or further crystallisation in the crystal bed. The fluid temperature was controlled by a heater/chiller bath (FP32/HL, Julabo), with heat-exchange oil flowing through the service side of the heat exchanger, and before entering the jacket of the recirculation vessel. The oil temperature was controlled based on the recirculating bath temperature. The solvent temperature was monitored with a Pt100 temperature probe.

The filter assembly ID-12 (7 mL) was designed to enable XCT and used for most trials. The filter assembly ID-35 (320 mL) was used for the scale up trials. Details on the assemblies are provided in Table I and Fig. 2c.

**Table I:** Data Related to the Two Filter Assemblies

Scale		Filter ID-35	Filter ID-12
Material		304 Stainless Steel	Nylon 6
Dimensions (mm)	H	334	60
	ID	35	12
Filtration area (mm^2)		962	113
Max. flow rate (L/h)		160	19
Filter paper pressure (bar)		0.06 to 1.14	0.10 to 1.93

In order to facilitate sampling of the solid material in the ID-35 filter chamber, a removable ‘insert’ was fabricated. This was designed for post-experimental cake protection and removal, with a vertical slit running up the side to allow for sampling at different cake.

The percolation pressure (*∆P*_*b*_) is defined as the pressure drop over the particle bed. More specifically, this equals the measured pressure at the inlet (*∆P*_*inl*_) of the fixed bed assembly (Druck DPI 104, General Electric) minus the calibrated pressure drop (*∆P*_*empty*_) of the empty assembly at the same flowrate as shown in Table I, section 1 in the ESI:

1 $$ \Delta  {P}_b=\Delta  {P}_{inl}-\Delta  {P}_{empty} $$

2 $$ \Delta  {P}_{empty}=55756\ {\rho}_l\ {\varphi}^2+143260\ \mu\ \varphi \varphi =0.893\ {10}^{-3}\kern0.5em {f}_{pump} $$

Where *∆P*_*empty*_ is the pressure of empty system (MPa), *φ* is the flow rate (m^3/s) and *f*_*pump*_ is the inverter frequency in Hz, *ρ*_*l*_ and *μ* are the density (m^3/kg) and viscosity (Pa s) of the liquid respectively.

#### Operating Procedure

The crystal suspension held at ~20 °C (as per section 2.1) was partially deliquored in a Buchner funnel using water jet vacuum pump with a low pressure drop over the filter (<0.20 bar *∆P*) to obtain a thick slurry, and the mother liquors (filtrate). Part of the slurry was transferred to the filter chamber giving an initial bed height of approximately 10–15 mm in ID-12 and 60–80 mm in the ID-35. The filter chamber was closed and fitted into the percolation rig.

The mother liquor was transferred to the jacketed vessel and the temperature set-point set to 20°C (the crystallisation end-point temperature). After temperature equilibration, the circulation pump was started at a low flowrate, and throughput gently increased until the required pressure drop was reached. The pressure over the percolation assembly was controlled by small adjustments of the pump flowrate during the first 3 to 10 min after which the system stabilised. Percolation continued for another 10 to 15 min after which the pump was stopped and the filter chamber purged with low pressure nitrogen (~0.2 bar) for 30 min to dry the cake. The XCT was executed at this point with samples were collected from the top and bottom of the bed after XCT. The collected samples were dried overnight at 35°C.

The above procedure was repeated for a range of inlet pressures (*∆P*_*inl*_) between 1 to 5.5 bar (as explained in section 2.2.1 and Table I in the ESI). After completion of the pressure experiments, the reference samples were obtained by following the procedure above without percolation in the ID-35 filter assembly with the remainder of the slurry.

### X-Ray Computed Tomography (XCT)

**Fig. 3:**
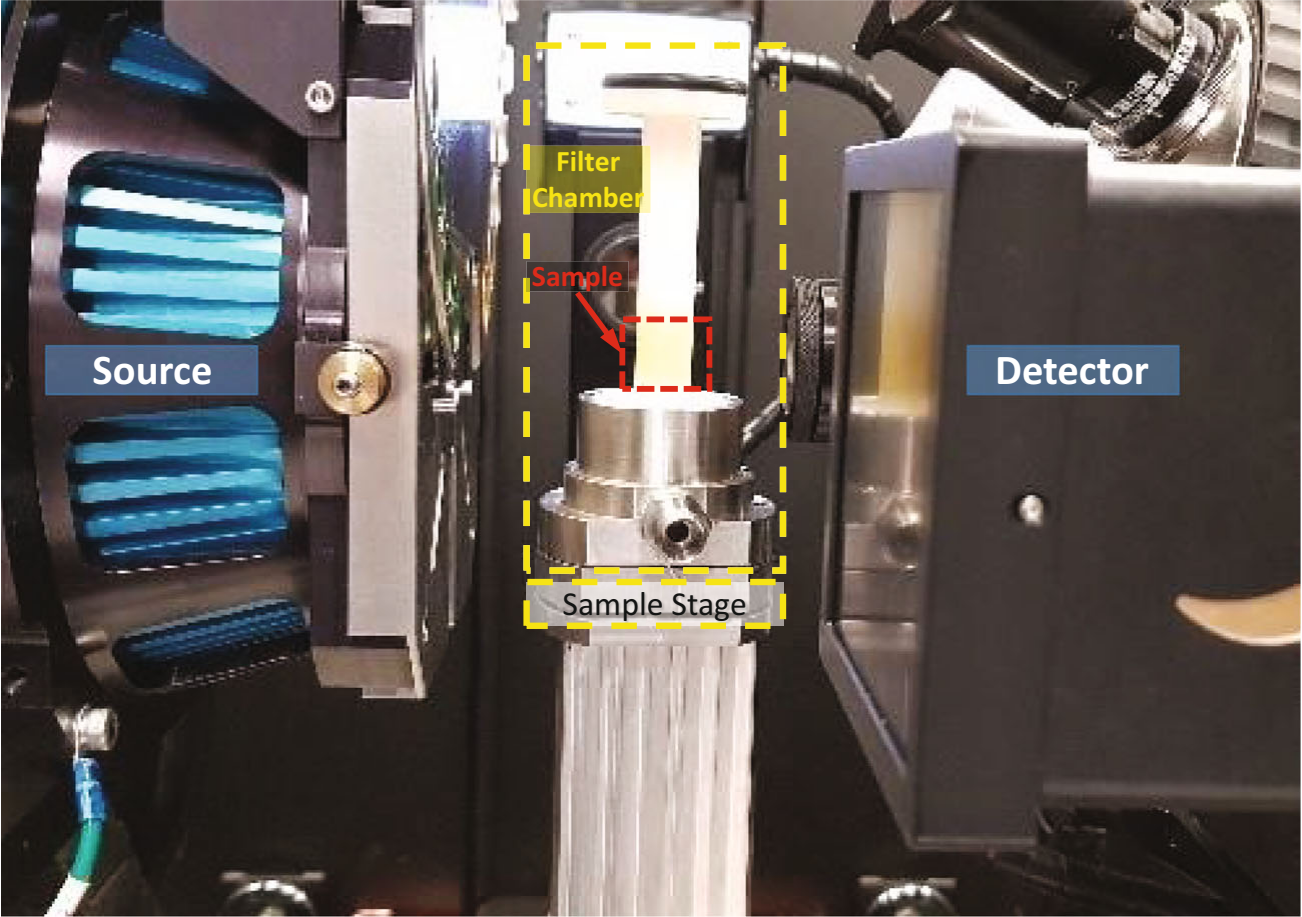
Small filter bed (ID-12) assembly fitted into the Xradia Versa 520.

PABA and LGA reference material and crystal beds after percolation at 1.5 bar, 3 bar and 4 bar were analysed using X-ray computed tomography (Xradia Versa 520, Zeiss) as shown in Fig. 3. Scans were performed on the bottom 4 mm (in height) section of the bed just above the filter cloth. For each scan, the voltage was 110 kV with 10 W of power. A 4x objective lens was used, the detector binning was 2x and the exposure time was 2 s per projection, with the source to sample and sample to detector distances of 50.04 mm and 31 mm, respectively. As the sample was rotated through 360 degrees, 1601 projections images were collected. The projection images were reconstructed using an implementation of the Feldkamp-Davis-Kress algorithm (25) in the proprietary Zeiss software to form a 3D image with a reconstructed voxel size of 4.17 μm.

The volumes were smoothed using a bilateral filter and the voidage fraction (*ε*) was calculated for each virtual cross-sectional slice of the 3D volume using a method adapted from (22), i.e. 1 minus the ratio between the area occupied by the powder and the total analysis area. Finally, slice to slice variations were smoothed using a robust linear regression over a window of 50 slices. A combination of Avizo 9.7 (Thermofisher Scientific, France) and MATLAB® (Mathworks, USA) were used for the visualisation and quantitative analysis.

### Particle Size and Shape Analysis

#### Optical Microscopy

Dry crystals were taken from the reference samples and observed on an optical microscope (Leitz DEAPLAN with a DeltaPix Indenio 55CIII camera) using standard 76 × 51 mm glass slide without a cover slip to protect the sample from breakage. LGA and PABA crystals were magnified 5x which provided optimal depth resolution. The smaller ibuprofen crystals were magnified 10x.

#### Particle Size Analysis

To detect subtle changes resulting from particle breakage a particle size analysis needs to be sensitive, robust, and reproducible. An automated optical microscope (Morphologi G3, Malvern Instruments Ltd.; UK) was used to measure the size and shape of individual dry particles. The Morphologi G3 has found significant application on a variety of different particle systems within the particle sizing and shape characterisation fields; typically with mineralogical and pharmaceutical based systems (26). To minimise agglomeration, stacking and breakage (27) a 13 mm^3^ sample was dispersed by a 0.5 bar ‘pulse’ of nitrogen to deposit the material over a 50 mm diameter circle. To balance capturing crystal details and maintaining good depth resolution the 5x and 10x magnification lenses were utilised according to the crystal size. This method was able to capture particles of 3 to 500 μm.

The G3’s reproducibility was assessed with five repeat measurements giving a variance between *F* curves of the size distribution of less than 5% (for full details see the ESI, section 2). After percolation at inlet pressure of 4 bar (*∆P*_*b*_=3.78 bar) the *F* curve shifts significantly outside this range confirming that the observed PSD changes are significant. In addition this shows the changes are due to the percolation process rather than fragmentation of crystals in the particle sizer as observed by Saifoori *et al*. (27).

The G3 uses a pixel based algorithm to approximate various crystal properties such as the shape, the length *L* and width *W* of each crystal as well as the circularity (*C*) and elongation (*E*) based on:

3 $$ Circularity:\kern8.5em C=\sqrt{\frac{4\pi }{P^2}{A}_{pix}} $$

4 $$ Elongation:\kern7.5em E=1-\frac{W}{L}=1-\alpha $$

With *P* is the length of the perimeter, *A*_*pix*_ is the particle area based on pixels and *α* is the inverse of the aspect ratio.

## Results

### Bed Structure

**Fig. 4:**
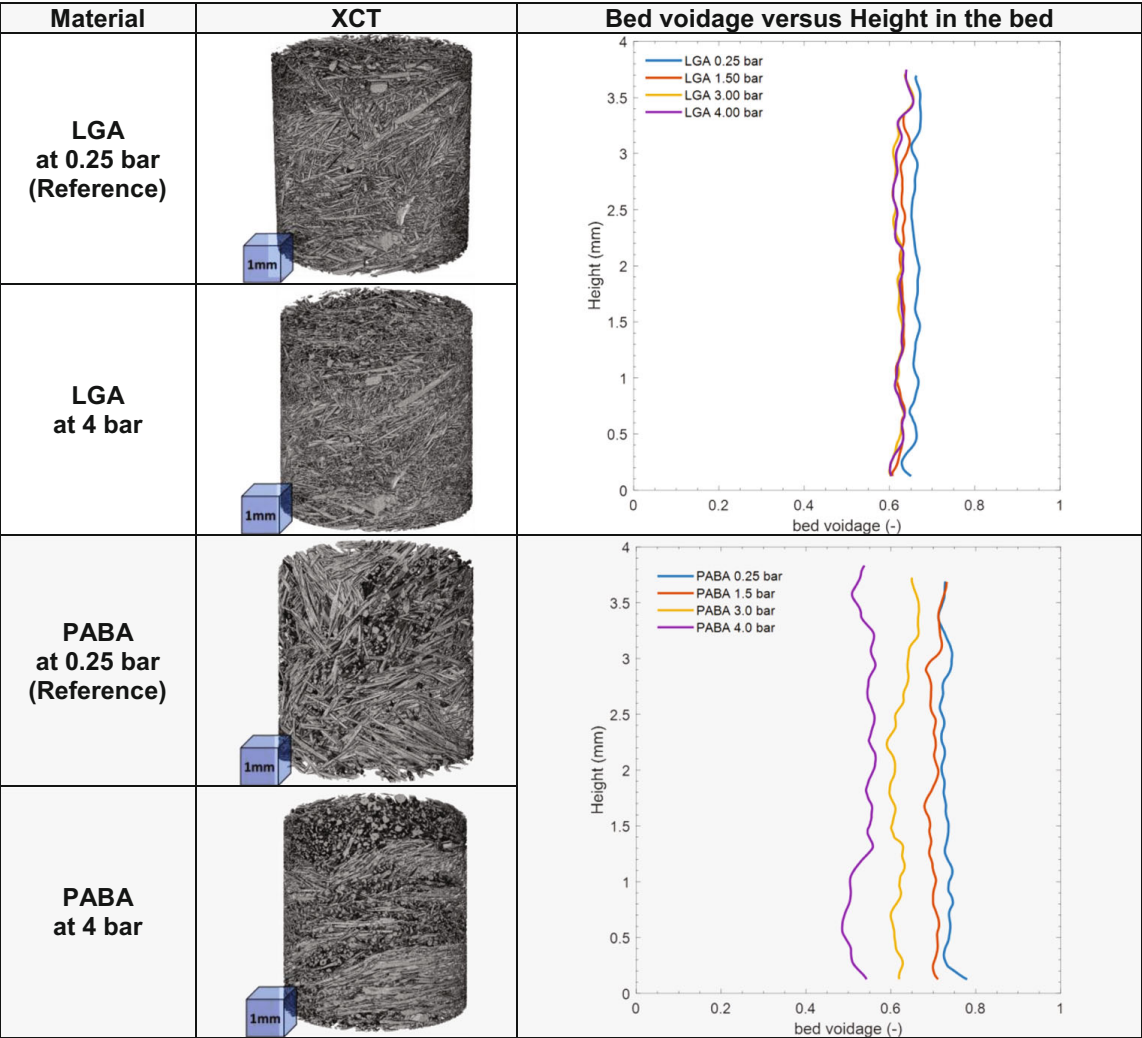
Visualisation of the bed structure and profiles of the bed voidage as a function of height for LGA and PABA. At different inlet pressures (*∆P*_*inl*_).

Qualitative and quantitative information on the bed structure was provided by XCT as shown in Fig. 4. Generally, there was significant compression of the bed as pressure increased. In the case of LGA large crystals are observed moving closer to the bottom of the bed and the length scale of crystals appears to shorten. The PABA crystals reorganise by rotating the major axis of the needles towards the horizontal plane, giving a layered appearance when percolated at inlet pressure of 4 bar (equal to *∆P*_*b*_= 3.92 bar (ibuprofen), 3.78 bar (LGA) and 3.22 bar (PABA)).

The bed voidage (*ε*) is the volume fraction of the bed unoccupied by solid and is a simple quantitative structural indicator of the crystal bed. The voidage as determined by XCT (Fig. 4) confirms the compression of the bed; the voidage for LGA decreases by 5% compared to the reference material, for PABA crystals voidage progressively decreases with increasing pressure. When pressure is applied over a bed of needle-shaped crystals, force is exerted on each crystal. If the magnitude of exerted forces on the crystals are sufficient, then the bed structure/voidage can change irreversibly by re-arrangement of particles. This so-called ‘cake collapse’ is observed clearly for PABA in Fig. 4b. At high percolation pressures, the forces can exceed the critical breakage stress of the particles resulting in crystal breakage, which further reduces the voidage. The PSDs in section 3.2 show this is indeed the case for both LGA and PABA.

The bed voidage is an important factor in the Ergun equation (28,29), which correlates the percolation pressure drop to the percolation flowrate:

5 $$ \Delta  {P}_b=150\frac{\mu {H}_{bed}}{d_s^2}\ \frac{{\left(1-\varepsilon \right)}^2}{\varepsilon^3}\ {v}_l+1.75\ \frac{\rho_l{H}_{bed}}{d_s}\frac{\left(1-\varepsilon \right)}{\varepsilon^3}\ {v}_l\left|{v}_l\right| $$

**Fig. 5:**
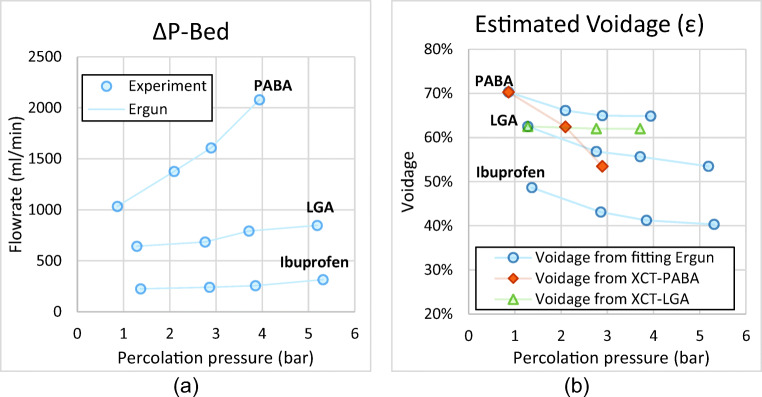
Estimation of the voidage using the Ergun equation: (**a**) the percolation flowrate as function of observed percolation pressure and (**b**) estimated bed voidage from Eq. 5 as function of pressure.

With the superficial liquid velocity (*v*_*l*_) obtained from measuring the percolation flowrate (*φ*). The percolation pressure and resulting flowrate are shown in Fig. 5a. At the same percolation pressure, the ibuprofen plates have the highest resistance to flow, with the large PABA needles having a significant lower resistance. From these we can obtain a second estimate of the voidage averaged over the whole bed. Assuming that reversible changes in voidage are negligible, the average voidage in the bed and the bed height are correlated as the total solid volume in the bed is constant:

6 $$ {H}_{bed}={H}_{bed.o}\frac{1-{\varepsilon}_o}{1-\varepsilon } $$

where *H*_*bed*. *o*_ and *ε*_*o*_ are the initial bed height and voidage, respectively. The value of the particle size *d*_*s*_ in the Ergun equation relates the area of solid spheres to their volume (*A*/*V* = 6/*d*_*S*_). For needle shaped particles with dimensions *L* × *W* × *H* the area to volume ratio and the resulting value of *d*_*s*_:

7 $$ \frac{A_s}{V_s}=\frac{6}{d_s}=2\frac{\left(\ L\ W+ LH+ WH\right)}{L\ W\ H}=2\left(\frac{1}{H}+\frac{1}{W}+\frac{1}{L}\right)\to {d}_s=\frac{3}{\frac{1}{H}+\frac{1}{W}+\frac{1}{L}} $$

The term 1/*L* may be considered negligible for high aspect ratio crystals. To estimate the crystal height *H* we calculated the voidage as function of *H* using Eq. 5 for the lowest flowrate and inlet pressure (*∆P*_*inl*_=0.25 bar). We then set *H* so that the voidage from Eq. 5 equals the voidage obtained from the XCT measurements for PABA and LGA. This resulted in *H*/*W*= 0.32 for PABA which compares well to observations with XCT. For LGA *H*/*W*= 0.11which is low compared to both the XCT data and observations from Hallac *et al*. (30) who found *H*/*W* ranges between 0.2 to 0.5. For ibuprofen *H*/*W*= 0.15 was estimated from SEM images.

The voidage for LGA determined from the XTC data (for the bottom of the bed) is constant at 62%, independent of pressure. When evaluating the voidage based on Eq. 5, it significantly falls. We think this behaviour is consistent with filter blinding. For PABA the XCT data indicates significant reduction of density in the bottom of the bed, whereas the overall voidage as determined from Eq. 5 remains high, indicting the upper regions of the bed do not compress as much as the bottom. This is consistent with the observation that PABA crystals don’t break near the top of the bed; (*L*/*L*_*ref*_)_*top*_ > 0.9. At 50%, the ibuprofen bed voidage is very low compared to the other two materials. It reduces a further 10% at percolation pressures up to *∆P*_*b*_= 2 to 3 bar. In section 3.3 we see that most breakage occurs below 2 bar which explains why there are only minor voidage changes above 2 bar.

### Visual Confirmation of Crystal Breakage after Percolation

**Fig. 6:**
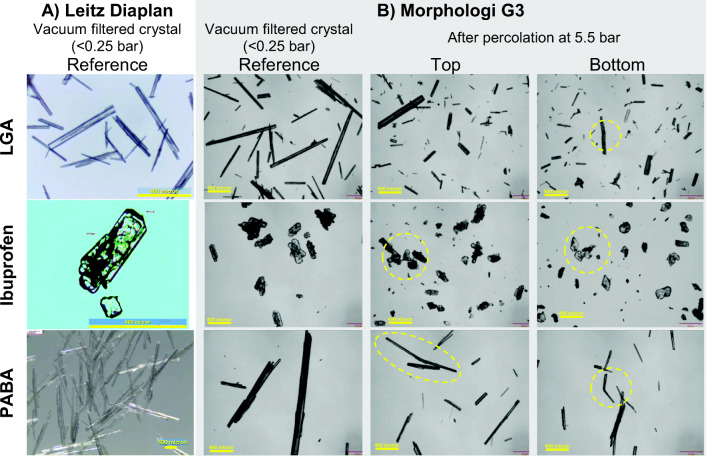
Observation of breakage in the three materials using: (**a**) the Leitz Diaplan optical microscope and (**b**) Morphologi G3, for the reference material and percolated samples at inlet pressure of 5.5 bar (top and bottom). The scale indicated by the yellow bar is 400 μm.

**Fig. 7:**
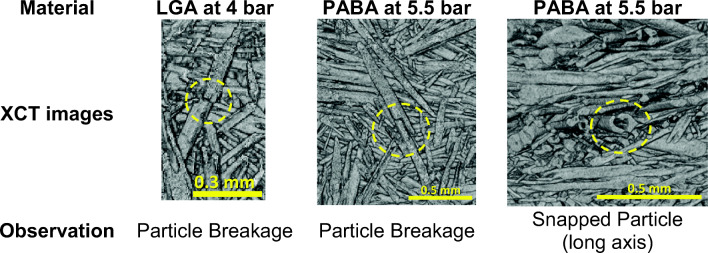
Examples of crystals breakage in a bed of crystals after percolation observed by XCT for LGA and PABA at different inlet pressures (*∆P*_*inl*_). The yellow dotted circles highlight fractures.

Typical changes between the reference sample of the crystal slurry and the samples after percolation at 5.5 bar (top and bottom) are shown in Fig. 6 (optical microscopy) and Fig. 7 (XCT). Before percolation, the LGA and PABA crystals have a well-defined straight needle shape, with smooth curved tips. Note that the PABA crystals appear to be hollow needles with a square cross section (Figs. 1d and 6). After percolation, the crystals are significantly shorter, and several have a ‘jagged’ end, indicative of breakage. Examples of ‘bent’ crystals two fragments have not completely disconnected are also seen in Fig. 6. Breakage is also detected in the XCT data (Fig. 7) which show that a fracture plane is normal to the major axis. We postulate that needle-shape crystals beak as result for bending along the major axis in response to forces exerted by crystals above and below it. When the critical breakage stress in the material is exceeded the crystals snap, much like raw spaghetti.

The ibuprofen crystals consist of clusters of thin extended hexagonal plates (thickness between 1 and 35 μm) formed by surface nucleation of new plates on existing growing plates. At high pressure, some clusters survive at the top of the bed, but at the bottom, where the forces are highest as particles support the entire bed above, the plates are completely fragmented.

### Confirmation of Breakage with 2D Volume Distributions

**Fig. 8:**
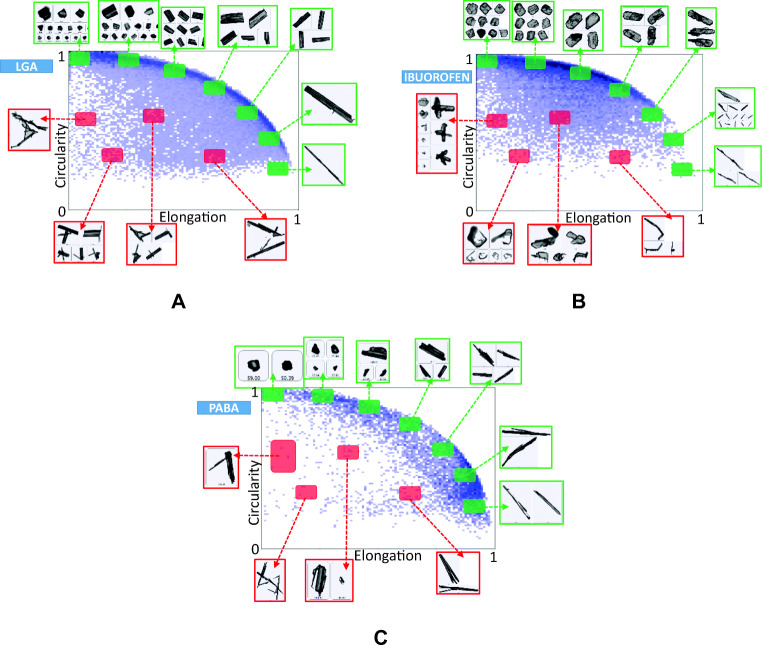
Typical G3 particle size data for reference samples of (**a**) LGA, (**b**) ibuprofen and (**c**) PABA. Highlighted areas with green colour represent combinations of (Elongation, Circularity) characteristic for single particles; red regions show clusters of overlapping particles for which the pixel based statistics are not representative of the individual particles.

To illustrate the nature of the particles observed we correlate circularity and elongation in Fig. 8. Each blue dot represents a single particle, or a cluster of particles, which is treated by the G3 as a single particle. Highlighted areas with green colour represent combinations of (*E*, *C*) characteristic for single particles. For crystals in these regions the particle area based on pixels equals to *A*_*pix*_ = *L* × *W*. The red regions show clusters of overlapping particles for which the pixel based statistics are not representative of individual particles (*A*_*pix*_ ≪ *L* × *W*) and should thus not be used in statistics describing particle distribution. A number of authors have used the Morphologi G3 alongside laser diffraction techniques in order to characterise particle shape factors (31–36). They confirm that the particle size measured by Morphologi G3 tends to be larger than those measured under diffraction due to the presence of overlapping particles (34,36). We developed a new algorithm to remove the impact of overlapping particles.

#### Filtering Algorithm Based on Observed Circularity and Elongation

To obtain more representative PSDs, the G3 software allows manual filtering, but this is time consuming and subject to operator bias and error. A filtering algorithm was devised based on a general description of high-aspect ratio particles. Consider a rectangle with rounded corners with radius *r* = *βW* ≤ *W*/2, a total length L and width *W* = *αL*, where *α* is the inverse of the aspect ratio and *β* determined the radius (*β*= 0–0.5). The circumference *P* and area *A* for such particle can be described as function of *α* and *β*:

8 $$ P=2\ \left(1+\alpha +\left(\pi -4\right)\ \alpha \beta \right)\ L $$

9 $$ A=\left(\alpha +\left(\ \pi -4\right){\left(\alpha \beta \right)}^2\right){L}^2 $$

The upper boundary of the circularity plot is well described as function of elongation when *β* = 0.5*α*^0.8^. In this case, the circularity as function of the inverse aspect ratio *α* is given by:

10 $$ {C}_{rnd}\left(\alpha \right)=\sqrt{\frac{4\pi A}{P^2}}=\sqrt{\pi \frac{\alpha +0.25\ \left(\ \pi -4\right)\ {\alpha}^{3.6}}{\ {\left(1+\alpha +0.5\left(\pi -4\right)\ {\alpha}^{1.8}\right)}^2}},\kern0.5em with\kern0.5em \alpha =1-E $$

**Fig. 9:**
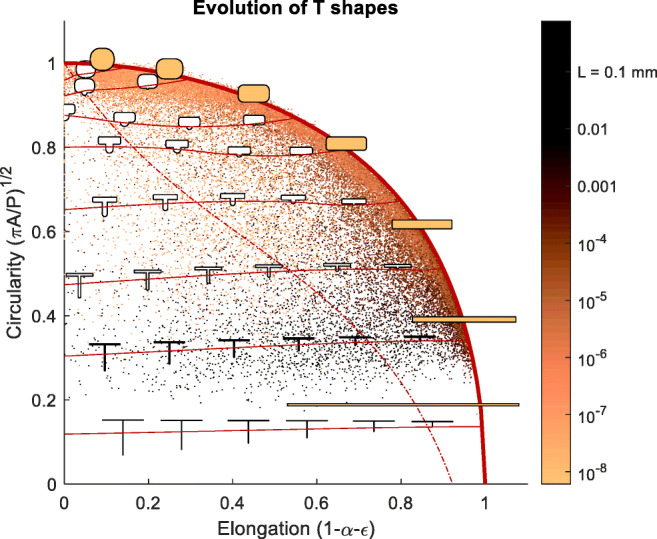
Evolution of T shapes with the length of the leg, *εL*. The outer fat solid line represents rounded rectangles (Eq. 10). The curved horizontal solid lines represents particles with the same *L* and *W* but *ε* between 0 and 1. The dashed line represents the boundary of the criterion in Eq. 15. Particles, represented by dots, below this line are excluded from the volume distribution.

This result suggests that longer particles with higher aspect ratios, have less rounded (or more regular) ends, and short particles are more rounded, i.e. for *α* = 1 the particle is a circle, and for *α* → 0 the particle becomes a long rectangle (Fig. 9).

As can be observed from Figs. 8 and 9, many particles do not lie on the outer edge described by Eq. 10, and far away from the outer edge the particles can be seen to be constructs of multiple particles. To investigate the effect of two particles overlapping, we simulate the second particle as the ‘leg’ of a ‘rounded T’ (see examples in Fig. 9). The leg has a length *εL* and sticks out at right angles from the base particle (the bar of the T). The elongation of such particle is:

11 $$ E=1-\frac{W+\varepsilon L}{L}=1-\alpha -\varepsilon $$

The T’s leg has a width *W*_*T*_ = min(*W*, *L* − *W*, *εL*) and is rounded off with a semi-circle of radius *W*_*T*_/2. When *εL* < *W* the “leg” is a semi-circle with diameter *εL*, essentially a small bump on an otherwise rectangular particle. If we define *α*_*T*_ by *W*_*T*_ = *α*_*T*_*L*, the circumference *P* and area *A* become:

12 $$ P/2L=1+\alpha +\left(\pi -4\right)\alpha \beta +\varepsilon +\left(\frac{\pi }{4}-\frac{3}{2}\right){\alpha}_T $$

13 $$ A/{L}^2=\alpha +\left(\ \pi -4\right){\left(\alpha \beta \right)}^2+2\left(\varepsilon -{\alpha}_T\right){\alpha}_T+\frac{\pi }{8}{\alpha}_T^2 $$

And the circularity of the two overlapping particles forming the T is:

14 $$ C=\sqrt{\frac{4\pi A}{P^2}}=\sqrt{\pi \frac{\alpha +\left(\ \pi -4\right){\left(\alpha \beta \right)}^2+2\left(\varepsilon -{\alpha}_T\right){\alpha}_T+\frac{\pi }{8}{\alpha}_T^2}{\ {\left(1+\alpha +\left(\pi -4\right)\alpha \beta +\varepsilon +\left(\frac{\pi }{4}-\frac{3}{2}\right){\alpha}_T\right)}^2}} $$

When the length of the second particle is changed by increasing *ε* from 0 to a number close to 1 keeping *α*_*T*_ = 1, the two overlapping particles combined have a significant lower elongation, but virtually no change in circularity. As such, the T shaped particles appear in locations (*E*, *C*) associated with overlapping particles. Also apparent is that rather small extrusions on a particle can result in particles moving a significant distance away from the smooth rounded rectangle described by Eq. 10.

Based on this analysis we defined a lower boundary below which we deem *W* to deviate too much from the actual value. This filter is based on *C*_*rnd*_(*α*) for the rounded particle from Eq. 10 and one additional term:

15 $$ {C}_{crit}\left(\alpha \right)={C}_{rnd}\left(\alpha \right)-0.5\left(1-{\mathrm{e}}^{-3\ \left(1-\alpha \right)}\right) $$

The filter criterion above removes a small number fraction of the particles (typically <4%), that have a disproportionate impact on the volume distributions as the solidity of the particles is very low. The volume of particles near the boundary is still an overestimate, but we found that tightening the criteria did not significantly alter the resulting volume distributions.

#### 2D Volume Distribution of the Reference Materials

The particles that lie above the exclusion criterion Eq. 15 are included in the volume distribution which is estimated based on the assumption that particle *i* is a cuboid with the height proportional to the width, *H* = *ξW*

16 $$ {V}_i=\xi\ {W}_i^2{L}_i $$

For the primary particles in freshly crystallised organic materials this is not unreasonable. The two dimensional volume distribution on an interval (*∆L*, *∆W*) can be estimated from Eq. 17:

17 $$ \frac{\partial^2{f}_V}{\partial W\partial L}\Delta  L\Delta  W=\frac{1}{\sum_i{W}_i^2{L}_i}\sum \limits_{\begin{array}{c}L\  to\ L+\Delta  L\\ {}W\  to\ W+\Delta  W\end{array}}{W}^2L $$

**Fig. 10:**
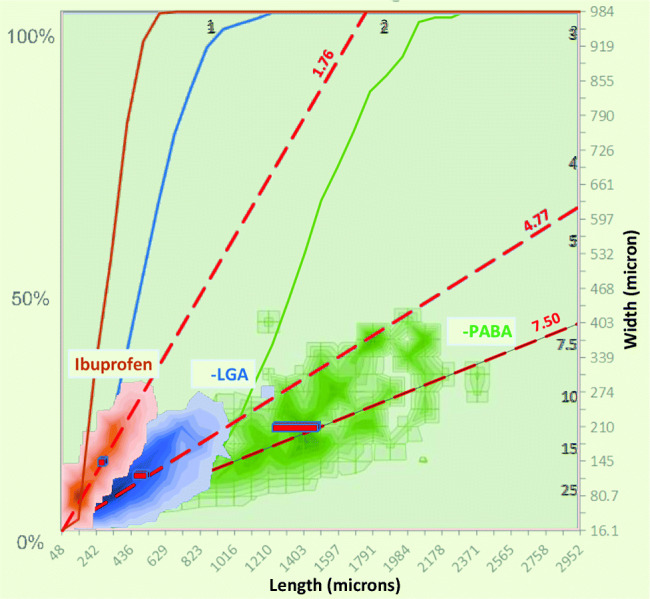
2D volume based size distribution of the three reference materials used in this study: PABA (*L*/*W*= 7.5), LGA (*L*/*W*= 4.8), and Ibuprofen (*L*/*W*= 1.8). The dashed red lines represent the volume-averaged aspect ratio. Also depicted are the cumulative volume distribution of *L* (Eq. 18, solid lines, left axis).

Therefore, if the assumption of constant *ξ* = *H*/*W* is valid for crystals with different widths *W*, its actual value does not affect the volume distribution (see ESI, section 5). The 2D particle size distributions of the three reference materials are shown in Fig. 10. Displayed is a contour plot of the volume distribution $\frac{\partial^2{f}_V}{\partial W\partial L}$ of particles that met the criterion in Eq. 15. Also included is the cumulative volume fraction *f*_*V*_(*L*):

18 $$ {f}_V(L)={\int}_0^L{\int}_0^{\infty}\frac{\partial^2{f}_V}{\partial W\partial L} dW\  dL\le 1 $$

The red rectangle, representing the centre point of the distribution (Figure 10), is scaled to, and located at, volume averaged length *L*_*av*_ and width *W*_*av*_ of the particles. Figure 10 shows the reference size distribution for the three materials; PABA has the longest crystals, up to ~2 mm long, with an aspect ratio of 7.5. LGA is smaller with a maximum length of ~0.825 mm, and an aspect ratio of 4.8. Ibuprofen is half the size of LGA with a low aspect ratio of 1.8. The combination of (*L*, *W*) for crystals of all three materials fall in well-defined regions that tapers to a point neat the origin, indicating that range of aspect ratios is the same at all scales. This suggests that the relative growth rates of the different crystal planes was constant.

#### The Impact of Percolation on 2D Volume Distributions

**Fig. 11:**
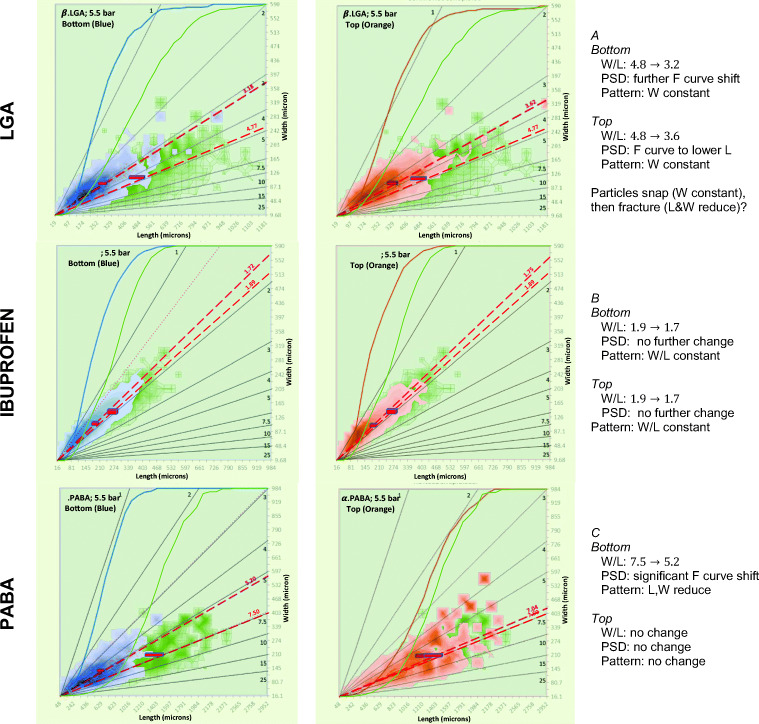
LGA, ibuprofen and PABA results before (reference, green) and after percolation at an inlet pressure of 5.5 bar (bottom of the bed in blue and top in orange). Solid grey lines represent constant aspect ratio *L*/*W* (number given, ≥1 by definition). Note, the contour drawing algorithm does not respect that *L*/*W* cannot be less than 1.

2D volume distributions (Eq. 18) for the three systems after percolation at *∆P*_*inl*_= 5.5 bar confirm that the observed breakage is assigned to both small and large crystals (Figure 11). For each component the PSD of samples from the bed after percolation at a set pressure are plotted over the distribution of the reference material. Each figure shows that the average size of particles reduces significantly when applying pressure. This effect is more pronounced for the samples taken from the bottom.

These observations are consistent with the reduction in voidage discussed in section 3.1, and show unequivocally that the three different materials experience a significant change in PSD after percolation. The ESI contains 2D size distributions for a range of inlet pressures for each material (section 7), from these we observe:

- **LGA** (Fig. 11a): needle-like crystals that snap at percolation pressures (*∆P*_*b*_) between 1.33 and 2.78 bar (*∆P*_*inl*_= 1.5 to 3 bar), resulting in a reduction in length, at constant width. At *∆P*_*b*_ over 2.78 bar, the average length reduces further, indicating further breakage.
- **Ibuprofen** (Fig. 11b): crystals consists of stacks of thin hexagonal plates that shatter at a *∆P*_*b*_< 1.42 bar (*∆P*_*inl*_<1.5 bar), resulting in unconnected plates of 1/3 to 2/3 of the length of the original plate. These smaller plates can resist further fracture. Interestingly, all plates, top and bottom break at once.
- **PABA** (Fig. 11c): crystals consist of long hollow needles that break at *∆P*_*b*_= 2.43 and 3.22 bar (*∆P*_*inl*_= 3.0 to 4.0 bar), causing a significant reduction in aspect ratio as the needles snap in two like the LGA crystals. However, just like the ibuprofen crystals, both *L* and *W* reduce. We postulate that the breakage of the four thin walls of a PABA crystal results in additional fragments over and above the needle shaped main breakage products. At higher pressures, more breakage is observed. The top of the PABA bed is not affected, indicating the force on crystals near the top remains too low for breakage.

In general, there appears to be a critical pressure below which little or no breakage occurs. As the pressure difference over the height of the bed increases, the bottom particles experience the highest forces, and break first. At the same location, more breakage occurs at higher pressures, though this effect diminishes once all crystals are below a critical size. Further we observe that needle shaped crystals (LGA, PABA) have daughter particles that are also needle shaped resulting in a significant reduction of *L* at constant *W*. Thin plate-like crystals (Ibuprofen, and the 4 walls of the hollow PABA crystal) on the other hand have irregular shaped daughter fragments causing both *L* and *W* to reduce.

#### Effect of Pressure on the Average Particle Length and Width

**Fig. 12:**
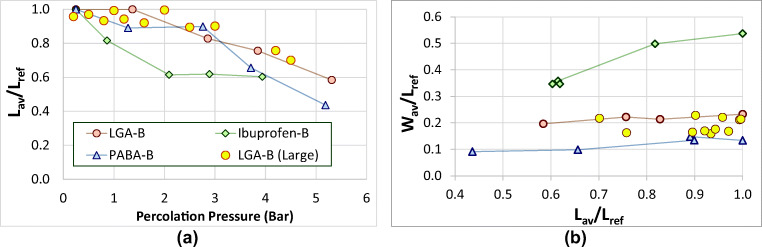
Evolution with percolation pressure of the average particle dimensions relative to the reference sample: (**a**) Change of average particle length relative to the reference sample, *L*_*av*_/*L*_*ref*_ (**b**) correlation between the average width and length relative to the reference sample.

The extent of breakage may be captured by comparison with the averaged length of the reference sample *L*_*ref*_. With increasing pressure, the ratio *L*_*av*_/*L*_*ref*_ reduces (Fig. 12a). Figure 12b shows the impact of breakage on the width of the particle. As more and more breakage occurs (and *L*/*L*_*ref*_ reduces further and further below 1) *W*/*W*_*ref*_ is practically constant for LGA and reducing only slightly for the hollow PABA crystals. The plate-like crystals of ibuprofen shatter, changing both length and width as is clearly visible in Fig. 12b.

A coarse extrapolation of *L*_*av*_/*L*_*ref*_ from high pressures to low pressure gives a rough estimate of the critical breakage pressure *∆P*_*b*_ when *L*_*av*_/*L*_*ref*_ approaches 1. For LGA *∆P*_*b*_= 1.5–2 bar, for ibuprofen *∆P*_*b*_= 0.5–1 bar and for PABA *∆P*_*b*_= 2–3 bar.

#### Effect of Scale on Observed 2D Volume Distribution

**Fig. 13:**
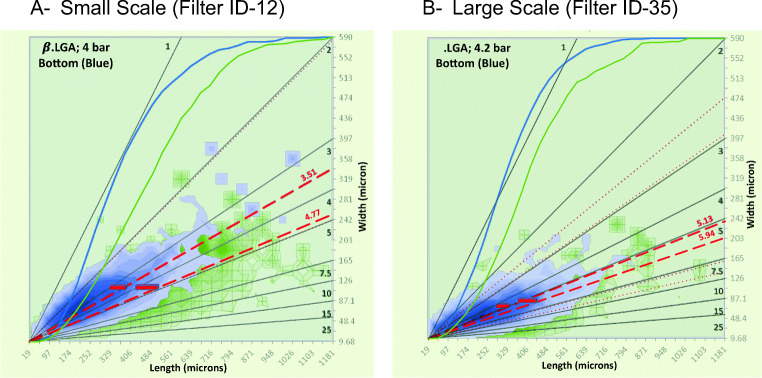
Comparison between PSDs obtained at (**a**) 12 mm bed diameter and (**b**) 35 mm bed diameter for LGA at an inlet pressure of 4 bar.

The ID-35 filter assembly contains a bed that is wider and higher than the ID-12 assembly. The 2D particle size distribution after percolation at *∆P*_*inl*_ of 4 bar for the both scales are compared in Fig. 13; significant breakage is observed, and in both cases the length is reduced indicating crystals “snap in two” at both scales. The change in *L*_*av*_/*L*_*ref*_ for the large scale percolation unit matched the small scale experimental data very well over the full range of applied pressures (Fig. 12a), and agree with the observations of an 80 mm scale pressure filter used by MacLeod and Muller (3), indicating the smaller, easy to operate percolation unit may be a versatile tool to assess the likelihood of crystal breakage.

## Discussion

The results of this work confirms high aspect ratio crystals break when the percolation pressure exceeds a critical value of *∆P*_*b*_, and shows that such breakage occurs across crystals at all length scales. MacLeod and Muller (3) developed a mechanistic model based on treating the crystals as cantilevers and estimating the force on the end of a crystal cantilever from the percolation pressure over the bed and the number of contact points between individual crystals. This model estimate the observed critical breakage stress of crystals, *σ*_*b*_ from the critical breakage pressure *ΔP*_*b*_ assuming the crystal is a cuboid cantilever (e.g. needles, plates):

19 $$ {\sigma}_b\approx 6\frac{\Delta  {P}_b.{L}_{ref}^{\raisebox{1ex}{$5$}\!\left/ \!\raisebox{-1ex}{$9$}\right.}}{{\left(1-{\varepsilon}_o\right)}^{\raisebox{1ex}{$11$}\!\left/ \!\raisebox{-1ex}{$9$}\right.}\ {W}_{ref}^{-\raisebox{1ex}{$2$}\!\left/ \!\raisebox{-1ex}{$9$}\right.}\ {H}_{ref}^{\raisebox{1ex}{$7$}\!\left/ \!\raisebox{-1ex}{$9$}\right.}} $$

The above assumes that crystals are solid, which is not the case for the hollow PABA crystals, so the breakage strength will be an underestimate for the true strength of PABA. Eq. 19 was applied on the data from the three materials (Table II), which shows that ibuprofen is the weakest material, and PABA the strongest. The value of *H*/*W* for LGA in this work ranges from 0.1 based on the relationship between percolation pressure and flow to 0.3 from the XCT work. MacLeod and Muller (3) found *H*/*W*=0.5 and Hallac *et al*. (30) found *H*/*W*= 0.2–0.5. The equivalent estimates of breakage strength ranges from 20 to 47 MPa. The 35 mm filter data is consistent with the small 12 mm filter, indicating successful scale down. These values compare well with the *σ*_*b*_(LGA) = 13–17 MPa found by MacLeod and Muller (3) in pressure filtration trials. Hallac *et al*. (30) used atomic force microscope (AFM) to break 63 cantilevers of LGA and found a median breakage strength of *σ*_*b*_(LGA) = 20 MPa with 90% of crystals having *σ*_*b*_(LGA) ≤ 45 MPa.

**Table II:** Estimates of the Breakage Strength and Correlation to the Volumetric Gibbs Free Energy

	∆P_*b*_ (bar)	*L*_*ref*_ (μm)	*W*_*ref*_ (μm)	${\frac{H_{ref}}{W_{ref}}}^{\ast }$	1 − *ε*_*o*_	*ρ ∆H*_*S* → *G*_ (kJ/L)	*ρ ∆G*_*S* → *G*_ (kJ/L)	*σ*_*b*_ (MPa)	Ref
LGA	1.5	464	108	0.10	0.33	1320	911	47.0	This work
				0.30				20.0	This work
Ibuprofen	1	257	138	0.15	0.49	580	228	8.86	This work
PABA	2.5	1355	200	0.32	0.26	1130	997	>54.7	This work
*Literature data*									
LGA						1320	911	13,17	MacLeod (3)
LGA						1320	911	9–49	Hallac (30)
Ibuprofen						580	228	8	Roberts (37)
Caffeine						668	353	10	Roberts (37)
Theophylline						1101	591	13	Roberts (37)
Aspirin						923	388	12	Roberts (37)
Paracetamol						985	520	13.38	Roberts (37)

^*^estimated in section 3.3 voidage

The critical pressure ∆P_b_(ibuprofen) = 1 bar is low, indicating breakage might occur even during gentle filtration. The resulting value of *σ*_*b*_(ibuprofen) = 8.86 MPa is similar to 7.7 MPa found by Roberts *et al*. (37) by extrapolating *σ*_*b*_(ibuprofen) for a series compacts with reducing voidage to zero voidage. We did not find equivalent data for PABA which has *σ*_*b*_ (PABA) > 54.7 MPa.

**Fig. 14:**
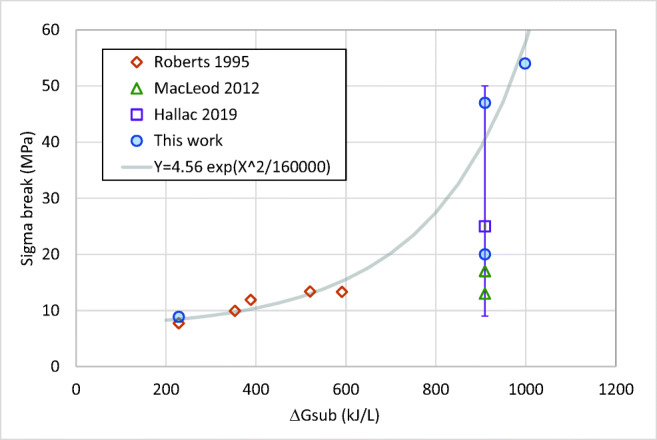
Correlations between observed breakage strength and the volumetric Gibbs free energy.

From a material science point of view, breakage across the needle axes involves breaking hydrogen-bonds (LGA, (22)), van der Waals forces (Ibuprofen, (23)) and pi-pi interactions (PABA, (24)). Our initial premise was that the nature of the bonding of molecules in their lattice correlates with the breakage strength. Initially we approximated the lattice energy with the heat of sublimation *∆H*_*S* → *G*_ expressed in kJ/L, which is indicative of the energy required to separate a volume (or plane) of molecules. This does not give a clear correlation. Including the entropic part of sublimation to give the volumetric Gibbs free energy of sublimation *∆G*_*S* → *G*_ (kJ/L) did allows correlating the data on 7 compounds as is shown in Fig. 14 and Table II (thermodynamic data and references are provided in the ESI). Accepting that the error in individual measurements are significant, the result over 7 data sets from literature in addition to the three data sets from this work indicates that when correlating breakage strength both lattice entropy and enthalpy need to be taken into the account.

## Conclusions

A detailed study of high aspect ratio crystals in filter beds shows that these break when the applied fluid pressure exceeds a critical pressure of 0.5–1 bar for ibuprofen, 1–2 bar for LGA and 2–2.5 bar for PABA. Experiments conducted in a continuous percolation rig combined with detailed size analysis shows two modes of breakage: (i) snapping of long crystals, resulting in reduction of average crystal length at constant width (PABA and LGA), and (ii) shattering of crystals resulting in reduction of both length and width (ibuprofen, PABA). The critical inlet pressure may be converted to a critical breakage stress. PABA is stronger than LGA, and ibuprofen is significantly weaker. The breakage strength of seven different compounds could be correlated to the volumetric Gibbs free energy though the error in the data is significant. The ID-12 and ID-35 mm diameter percolation assemblies give similar critical breakage strengths, and compare well to breakage literature data an 80 mm filter. The smaller, easy to operate percolation unit is thus a versatile tool to assess the likelihood of crystal breakage in large scale filtration operations.

### ACKNOWLEDGMENTS AND DISCLOSURES

We thank Kevin Roberts for discussions related to material selection and material science. The work was funded by the Advanced Manufacturing Supply Chain Initiative ‘Advanced Digital Design of Pharmaceutical Therapeutics’ (ADDoPT) project (Grant No. 14060), EP/N025075/1, AstraZeneca and the EPSRC. The scans were performed in the Henry Moseley X-ray Imaging Facility (HMXIF) at the University of Manchester, which was established through EPSRC grants EP/F007906/1, EP/I02249X/1 and EP/F028431/1. HMXIF is a part of the Henry Royce Institute for Advanced Materials, established through EPSRC grants EP/R00661X/1, EP/P025498/1 and EP/P025021/1.

## Electronic supplementary material

ESM 1 (PDF 5967 kb)
