# Supplementary material for: On the Breakage of High Aspect Ratio Crystals in Filter Beds under Continuous Percolation
Source: Pharm Res. 2020 Oct 29;37(12):231. doi: 10.1007/s11095-020-02958-x (PMC7596000; doi:10.1007/s11095-020-02958-x)
Supplement: Supplementary file 1 — (PDF 5967 kb) [file 11095_2020_2958_MOESM1_ESM.pdf]

On the breakage of high aspect ratio crystals in filter beds under continuous percolation

F.M. Mahdi(1), A.P. Shier(1), I.S. Fragkopoulos(1), J. Carr(2), P. Gajjar(2) and F.L. Muller(1,*)

(1)School of Chemical and Process Engineering, University of Leeds, Leeds LS2 9JT, UK

(2)Henry Moseley X-ray Imaging Facility, Henry Royce Institute for Advanced Materials, Department of Materials, The University of Manchester, Manchester, M13 9PL, UK

(Corresponding author: f.l.muller@leeds.ac.uk)

Electronic supplementary information (ESI)

1. Flow rate and pressure calibration

The flow rate provided by the pump can be accurately determined from the inverter frequency; this requires a calibration of the pump. This is performed under several conditions to note variations in flow/resistance of the filter. The pump has been calibrated using five different frequencies: 5, 15, 20, 30 and 40 Hz with two different scenarios: without and with filter cake (glass beds of two different sizes (D_{50}): 80 and 200 micron). The results shows that the pump flow rate with and without particles bed is the same. Also, it produces a linear trend in which pump delivery or liquid flow is directly proportional to the inverter frequency ($y = 53.59x$), whether the filter cake is present or not. Table 1 shows the experimental conditions as well as the system and

pump calibration. Percolation pressure (ΔP_b, over cake) is the difference between inlet pressure (ΔP_{inl}) and empty system pressure (ΔP_{empty}) with filter paper without cake.

Table 1: experimental condition: empty system (ΔP_{empty}), inlet (measured, ΔP_{inl}) and percolation pressure(ΔP_b, bar) as well as the flow rate (ml/min) for each material at different pump inverter (Hz)

Materials	Pump Inverter (Hz)	Flow Rate (ml/min)	Measured ΔP_{inl} (Bar)	Empty System ΔP_{empty} (Bar)	Percolation ΔP_b (Bar)
Ibuprofen	4.2	225	1.5	0.08	1.42
	4.5	241	3.0	0.08	2.92
	4.8	257	4.0	0.08	3.92
	5.9	316	5.5	0.13	5.37
LGA	12.0	643	1.5	0.17	1.33
	12.8	686	3.0	0.22	2.78
	14.8	793	4.0	0.22	3.78
	15.8	847	5.5	0.30	5.20
PABA	19.3	1034	1.5	0.43	1.07
	25.7	1377	3.0	0.57	2.43
	30.0	1608	4.0	0.78	3.22
	38.8	2079	5.5	1.13	4.37

The pump frequency (Hz) has converted into flowrate (g/min) using the pump calibration curve that we have made experimentally. The percolation pressure is the measured inlet filter pressure with crystals minus the one without crystals. In order to find the corrected net pressure, a square pressure (bar) has plotted against the flow rate (g/min) and the liner relationship of data has been used. The corrected net pressure has plotted vs. the flow rate as shown in the Results section (main manuscript) which shows that in all three cases the flowrate is proportional to the square root of the pressure, as predicted by the Ergun equation. Given the high flowrates (give in mm/s) and low bed heights (give in mm) giving liquid residence times in the order of ms.

The percolation pressure (ΔP_b), the pressure drop over the particle bed only, is obtained from measured pressure drop over the fixed bed assembly (ΔP_{tot}), minus the calibrated pressure drop (ΔP_{empty}) of the empty assembly at the same flowrate:

$$\Delta P_b = \Delta P_{tot} - \Delta P_{empty} \qquad \text{Eq. 1}$$

$$\Delta P_{empty} = 55756\,\rho_l\,\varphi^2 + 143260\,\mu\,\varphi \quad MPa \qquad \text{Eq. 2}$$
$$\varphi = 0.893\,10^{-3}\ f_{pump}\ {m^3}/{s}$$

Where f_{pump} is the inverter frequency in Hz.

2. Particle size and shape analysis

2.1 Characterisation of reference materials

Figure 1 shows images of dry crystals the crystals where captured using: Leitz Diaplan optical microscope (Leica Microsystems GmbH; Germany) and the G3 Morophologi. This figure show that LGA crystals consist of 0.1-0.9 mm long needles, Ibuprofen crystals consist of thin hexagonal plates (thickness between 1 and 35 micron) and PABA crystals consist of 0.6-2.5 mm long needles.

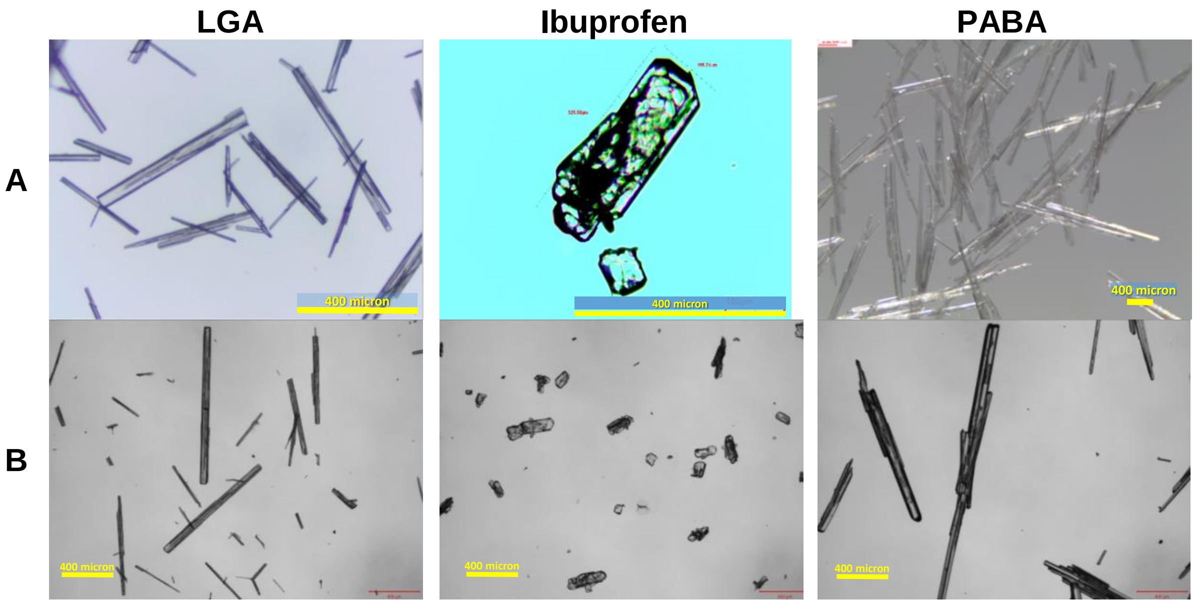

Figure 1: Light microscopy of the reference samples: A) Leitz Diaplan optical microscope and B) the Malvern G3 Morophologi

2.2 Particle size analysis

Once scanned by the instrument, particle parameters are calculated based on the pixelated image, so for perfectly rectangular particles only W and L can be identified.

For the bulk of the size distribution analysis, the circle equivalent diameter, d_{CE}, will be taken as a means of comparing the particle size. The value of d_{CE} (Eq. 3) results from the area of pixels $A_{pix} = number\ of\ pixels\ \times\ area\ per\ pixel$:

$$d_{CE} = \sqrt{\frac{4A_{pix}}{\pi}} = \sqrt{\frac{4L\,W}{\pi}} \qquad \text{Eq. 3}$$

To test the reproducibility of the measurements, 5 identical runs of beta-glutamic acid crystals after percolation at 4bar (Bottom sample) were compared, loading new crystals for each measurement. To compare the 5 curves we averaged the area fraction of particles as determined from the microscopic images by the Malvern G3 in each size bin i resulting in the average area fraction $\overline{f_i}$ and its standard deviation $\sigma_{f.i}$:

$$\overline{f_i} = \frac{1}{5}\sum_{j=1}^{5} v\%_{i,j}\,, \qquad \sigma_{f.i} = \sqrt{\frac{1}{5-1}\sum_{j=1}^{5}\left(f_{i,j} - \overline{f_i}\right)^2}\,, \qquad \text{Eq. 4}$$

And similar for the cumulative distribution

$$\overline{F_i} = \frac{1}{5}\sum_{j=1}^{5} F_{i,j}\,, \qquad \sigma_{F.i} = \sqrt{\frac{1}{5-1}\sum_{j=1}^{5}\left(F_{i,j} - \overline{F_i}\right)^2} \qquad \text{Eq. 5}$$

Figure 2 shows an average value of cumulative and frequency distributions with error bars indicating the standard deviation. The maximum value of $\sigma_{f.i}$ is ±11% of bin volume and $\sigma_{F.i}$ =±4.9% of cumulative volume. Also plotted is the distribution of the reference sample, demonstrating that the percolation at 4 bar results in a significant reduction of the PSD that is 2-4 times larger than the standard deviation. This confirms that the observed particle size changes are due to the percolation process rather than fragmentation of crystals in the particle sizer. The result for the d_{CE} evidence breakage on percolation, but provides no insight as to what really happens to the high aspect ratio particles.

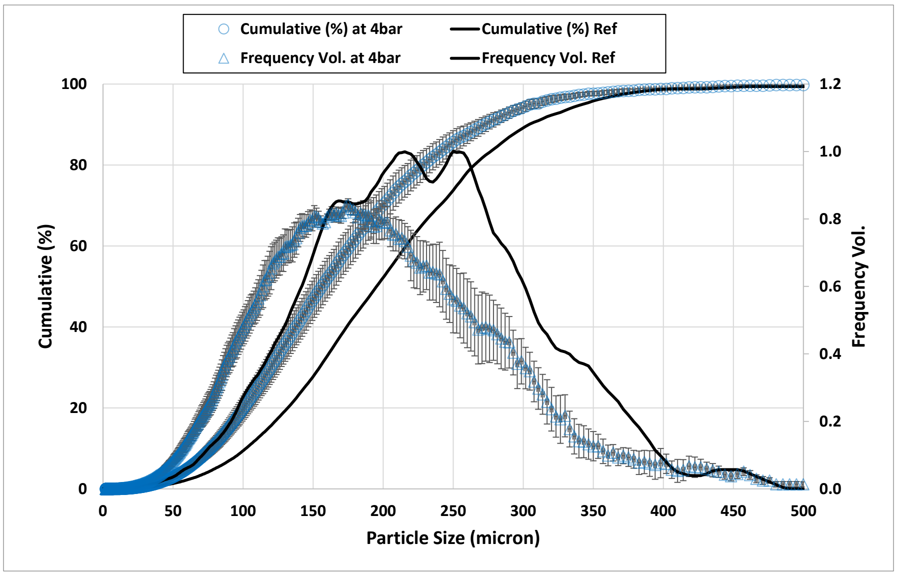

Figure 2: an average PSDE with standard deviation (error bars) using Morphology G3 for 5 separate measurements of percolated samples (bottom) for LGA at inlet pressure of 4 bar (ΔP_b =3.78 bar) and for the reference (Ref) material.

3. Bed Voidage

Table 2: data used to estimate the bed voidage

Material	Flowrate (Hz)	ΔP (bar)	Qv (ml/min)	ΔPsys (bar)	ΔP-Bed (bar)	eps XRT	xtalsolv	rho.l (kg/m3)	mu.l (Pa)	Lref	Wref	H/W	W	L/Lref	Ds	H.bed.init	eps.init	eps Ergun	ΔP-Ergun
**Ibuprofen**	4.20	1.50	225	0.13	1.37		45% EtOH	900	0.0023	257	138	0.15	138	0.9	54.0E-6	0.015	49%	49%	1.37
**Ibuprofen**	4.50	3	241	0.14	2.86		45% EtOH	900	0.0023	257	138	0.15	92	0.61	54.0E-6	0.015	49%	43%	2.86
**Ibuprofen**	4.80	4	257	0.15	3.85		45% EtOH	900	0.0023	257	138	0.15	116	0.62	54.0E-6	0.015	49%	41%	3.85
**Ibuprofen**	5.90	5.50	316	0.19	5.31		45% EtOH	900	0.0023	257	138	0.15	104	0.69	54.0E-6	0.015	49%	40%	5.31
**LGA**	12	1.50	643	0.22	1.28	0.64	water	1000	0.0010	464	108	0.102	108	1	30.0E-6	0.015	64%	64%	1.28
**LGA**	12.8	3	686	0.24	2.76	0.58	water	1000	0.0010	464	108	0.102	109	0.83	30.0E-6	0.015	64%	58%	2.76
**LGA**	14.8	4	793	0.29	3.71	0.58	water	1000	0.0010	464	108	0.102	107	0.76	30.0E-6	0.015	64%	57%	3.71
**LGA**	15.8	5.50	847	0.31	5.19		water	1000	0.0010	464	108	0.102	91	0.58	30.0E-6	0.015	64%	55%	5.19
**PABA**	19.3	1.50	1034	0.63	0.87	0.7	30%EtOH	850	0.0020	1355	200	0.318	203	0.96	48.3E-6	0.015	70%	70%	0.87
**PABA**	25.7	3	1377	0.91	2.09	0.65	30%EtOH	850	0.0020	1355	200	0.318	192	0.9	48.3E-6	0.015	70%	66%	2.09
**PABA**	30	4	1608	1.11	2.89	0.55	30%EtOH	850	0.0020	1355	200	0.318	193	0.66	48.3E-6	0.015	70%	65%	2.89
**PABA**	38.8	5.50	2079	1.56	3.94		30%EtOH	850	0.0020	1355	200	0.318	124	0.44	48.3E-6	0.015	70%	65%	3.94

4. Data filtering

The G3 software allows manual drawing of the polygons to filter the data, but is subjected to operator bias and error as well as time consuming. We devices a filtering algorithm based on exported text files containing, L, W, C and E data files.

As general description of a high-aspect ratio particle we use a rectangle with rounded corners such that for a particle has a total length L and width $W = \alpha L$ and rounded

corners with radius $r = \beta W \leq W/2$. The elongation for such particle is the same as for a rectangle $E = 1 - \alpha$. The circumference P and area A are (Eq. 6 and Eq. 7):

$$P = 2(W - 2r) + 2(L - 2r) + \ 2\pi r = (2\alpha(1 - 2\beta) + 2(1 - 2\alpha\beta) + \ 2\pi\alpha\beta)L \qquad \text{Eq. 6}$$

$$P = (2\alpha - 4\alpha\beta + 2 - 4\alpha\beta + \ 2\pi\alpha\beta)L = (2 + 2\alpha + (2\pi - 8)\alpha\beta)L$$

$$= 2\ (1 + \alpha + (\pi - 4)\ \alpha\beta)\ L \qquad \text{Eq. 7}$$

$$A = L(W - 2r) + 2r(L - 2r) + \ \pi r^2$$

$$A = \left((\alpha - 2\alpha\beta) + 2\alpha\beta(1 - 2\alpha\beta) + \ \pi(\alpha\beta)^2\right) L^2 \qquad \text{Eq. 8}$$

$$A = (\alpha + (\ \pi - 4)(\alpha\beta)^2)L^2 \qquad \text{Eq. 9}$$

Finally, the circularity based on P and A follows (Eq.1x):

$$C_{rnd}(\alpha, \beta) = \sqrt{\frac{4\pi A}{P^2}} = \sqrt{4\pi \frac{\alpha + (\ \pi - 4)(\alpha\beta)^2}{4\ (1 + \alpha + (\pi - 4)\ \alpha\beta)^2}} = \sqrt{\pi \frac{1/\alpha + (\ \pi - 4)\beta^2}{(1/\alpha + 1 + (\pi - 4)\ \beta)^2}} \qquad \text{Eq. 10}$$

By trial and error, we found that for $\beta = 0.5\alpha^{0.8}$ the edge of the circularity plot is well described (see Figure 3). In this case, the circularity as function of the inverse aspect ratio α is given by:

$$C_{rnd}(\alpha) = \sqrt{\frac{4\pi A}{P^2}} = \sqrt{\pi \frac{\alpha + 0.25\ (\ \pi - 4)\ \alpha^{3.6}}{(1 + \alpha + 0.5(\pi - 4)\ \alpha^{1.8})^2}}, \qquad with\ \alpha = 1 - E \qquad \text{Eq. 11}$$

This result suggest that particles with lower aspect ratios have more rounded (or less regular) ends.

As can be observed from Figure 3 and Figure 4, many particles do not lie on the outer edge described by Eq. 11, and far away from Eq. 11 the particles can be seen to be constructs of multiple particles. To investigate such constructs we add a rounded ‘leg’

to the rounded rectangle, thus forming a ‘rounded T’. The ‘leg’ of the ‘T’ sticks out at right angles from the base particle, and has a length εL. The elongation of such particle is therefore:

$$E = 1 - \frac{W + \varepsilon L}{L} = 1 - \alpha - \varepsilon \qquad \text{Eq. 12}$$

The T’s leg has a width $W_T = \min(W, L - W, \varepsilon L)$ and is rounded of with a half circle of radius $W_T/2$ and. When $\varepsilon L < W_T$ the “leg” is a half circle with diameter εL, essentially a small bump on an otherwise rectangular particle. Several example particles are provided in Figure 3 and Figure 4. If we define α_T by $W_T = \alpha_T L$, the circumference P and area A are:

$$P = 2(W - 2r) + 2(L - 2r) + \ 2\pi r - W_T + 2(\varepsilon L - W_T) + \frac{\pi}{2} W_T$$

$$\frac{P}{2L} = (\ \alpha - 2\ \alpha\beta) + (1 - 2\alpha\beta) + \ \pi\alpha\beta - \frac{\alpha_T}{2} + (\varepsilon - \alpha_T) + \frac{\pi}{4}\alpha_T \qquad \text{Eq. 13}$$

$$P/2L = 1 + \alpha + (\pi - 4)\alpha\beta \ + \varepsilon + \left(\frac{\pi}{4} - \frac{3}{2}\right)\alpha_T \qquad \text{Eq. 14}$$

$$A/L^2 \ = \alpha + (\ \pi - 4)(\alpha\beta)^2 \ + 2(\varepsilon - \alpha_T)\alpha_T + \frac{\pi}{8}\alpha_T^2 \qquad \text{Eq. 15}$$

Circularity:

$$C = \sqrt{\frac{4\pi A}{P^2}} = \sqrt{\pi \frac{\alpha + (\ \pi - 4)(\alpha\beta)^2 \ + 2(\varepsilon - \alpha_T)\alpha_T + \frac{\pi}{8}\alpha_T^2}{\left(1 + \alpha + (\pi - 4)\alpha\beta \ + \varepsilon + \left(\frac{\pi}{4} - \frac{3}{2}\right)\alpha_T\right)^2}} \qquad \text{Eq. 16}$$

To investigate the effect if the extend of overlap, we ‘grow a leg’ by increasing ε from 0 to a number close to 1. Figure 3 shows that this results in significant reduction in elongation, with virtually no change in circularity, and particles appear in locations associated with overlapping particles. Also apparent is that rather small extrusions on

a particle can result in particles moving a significant distance away from the smooth rounded rectangle described by Eq. 11.

Based on this analysis we defined a lower boundary below which we deem particles observed W to deviate too much from the actual value. This filter is based on Eq.06 for the rounded particle and one additional term as shown in Eq. 17.

$$C_{crit}(\alpha) = C_{rnd}(\alpha, \beta = 0.5) - 0.5\left(1 - \mathrm{e}^{-3\,(1-\alpha)}\right) \qquad \text{Eq. 17}$$

The filter criterion above removes a small number fraction of the particles (typically <4%), that have a disproportionate impact on the volume distributions as the solidity of the particles is very low. The volume of particles near the boundary is still an overestimate, but we found that tightening the criteria did not significantly alter the resulting volume distributions.

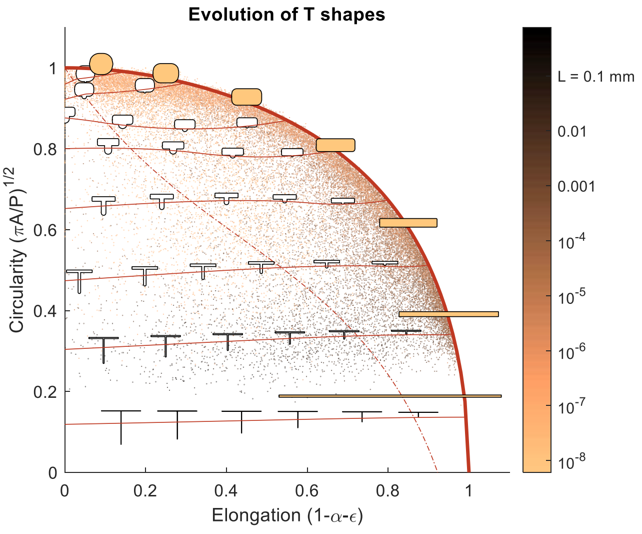

Figure 3: Evolution of T shapes when the length of the leg, εL. The outer fat solid line representes rounded rectangles (Eq. 11). The curved horizontal solid lines represents particles with the same L & W, but ε between 0 and 1. The dashed line represents the boundary of the criterion in Eq. 17. Particles below this line are excluded from the volume distribution.

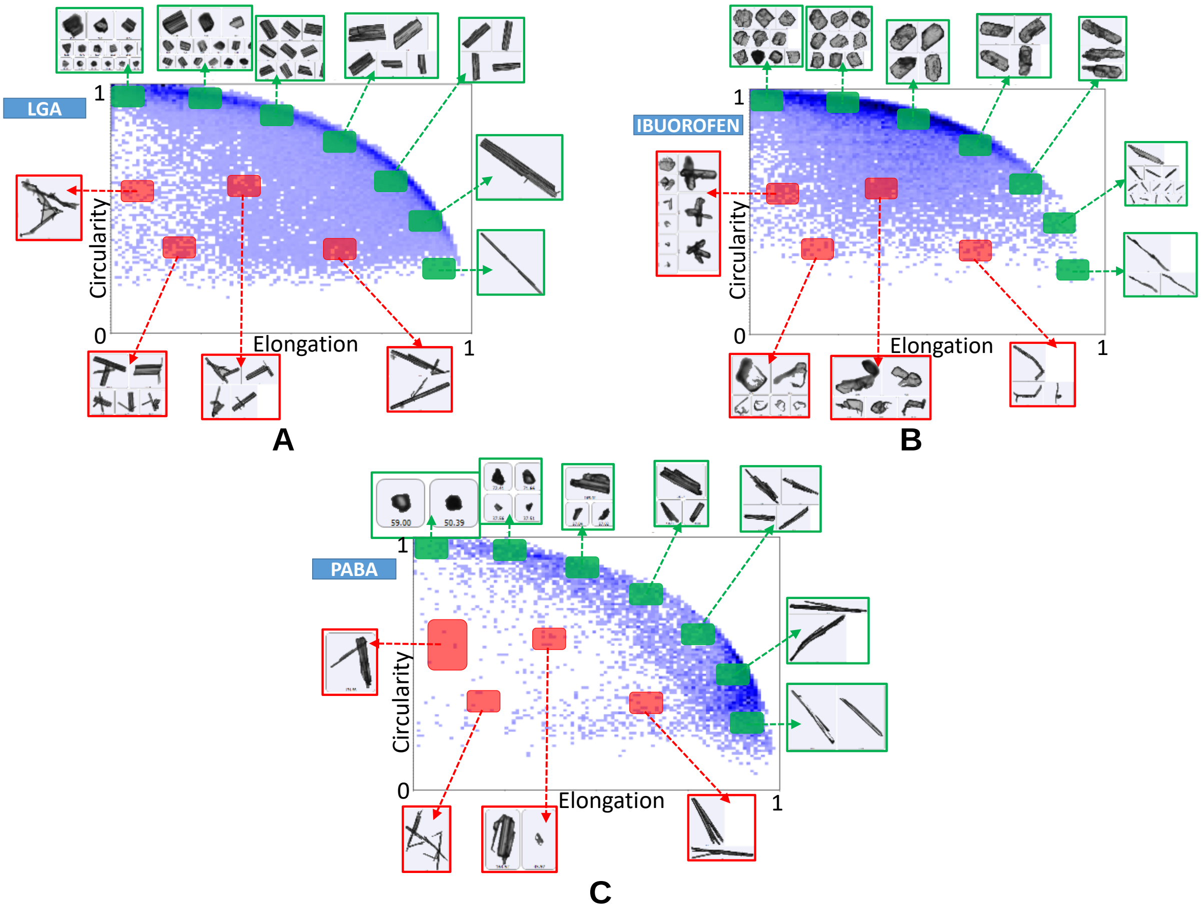

Figure 4: Typical G3 particle size data for reference samples of (A) LGA, (B) Ibuprofen and (C) PABA. Particles are displayed in with according to circularity vs. elongation. Areas of the distribution are highlighted with an image given to exemplify typical particles at that location. Highlighted areas with green colour represent combinations of (E,C) characteristic for single particles; red regions show clusters of overlapping particles for which the pixel based statistics are not representative of the individual particles and should thus not be used in statistics describing particles

5. 2D volume distribution of the reference materials

The particles that lie above the exclusion criterion demarqued by Eq. 17 are included in the volume distribution which is estimated based on the assumption that particle i is rectangular with the height proportional to the width, $H = \xi W$

$$V_i = \xi \, W_i^2 L_i \qquad \text{Eq. 18}$$

Such assumption is only warranted if backed up by visual inspection. For the primary particles in freshly crystallised organic materials this is however not unreasonable and

ξ is highly likely to be significantly less then 1. The two dimensional volume distribution on an interval $(\Delta L\ ,\ \Delta W)$ can be estimated from Eq. 19:

$$\frac{\partial^2 f_V}{\partial W \partial L} \approx \frac{1}{\sum_i V_i} \sum_{\substack{L\ to\ L+\Delta L \\ W\ to\ W+\Delta W}} \frac{V_{L,W}}{\Delta L \Delta W} = \frac{1}{\sum_i \xi\ W_i^2 L_i} \sum_{\substack{L\ to\ L+\Delta L \\ W\ to\ W+\Delta W}} \frac{\xi\ W^2 L}{\Delta L \Delta W}$$
$$= \frac{1}{\sum_i W_i^2 L_i} \sum_{\substack{L\ to\ L+\Delta L \\ W\ to\ W+\Delta W}} \frac{W^2 L}{\Delta L \Delta W}$$ Eq. 19

Therefore, as long as the assumption of constant ξ is valid, its actual value does not impact on the volume distribution as it divides out. The crystals size information obtained from the morphology G3 for PABA, LGA and Ibuprofen is shown in Figure 5. Displayed is a contour plot of the volume distribution $\frac{\partial^2 f_V}{\partial W \partial L}$ of particles that met the criterion in Eq. 17. Also included is the cumulative volume fraction $f_V(L)$:

$$f_V(L) = \int_0^L \int_0^\infty \frac{\partial^2 f_V}{\partial W \partial L} dW\ dL\ \leq 1$$ Eq. 20

The centre point of the distribution (red “particle”) is scaled to, and located at, the volume average mean length and width. Note, the contour drawing algorithm does not respect that L/W cannot be less than 1.

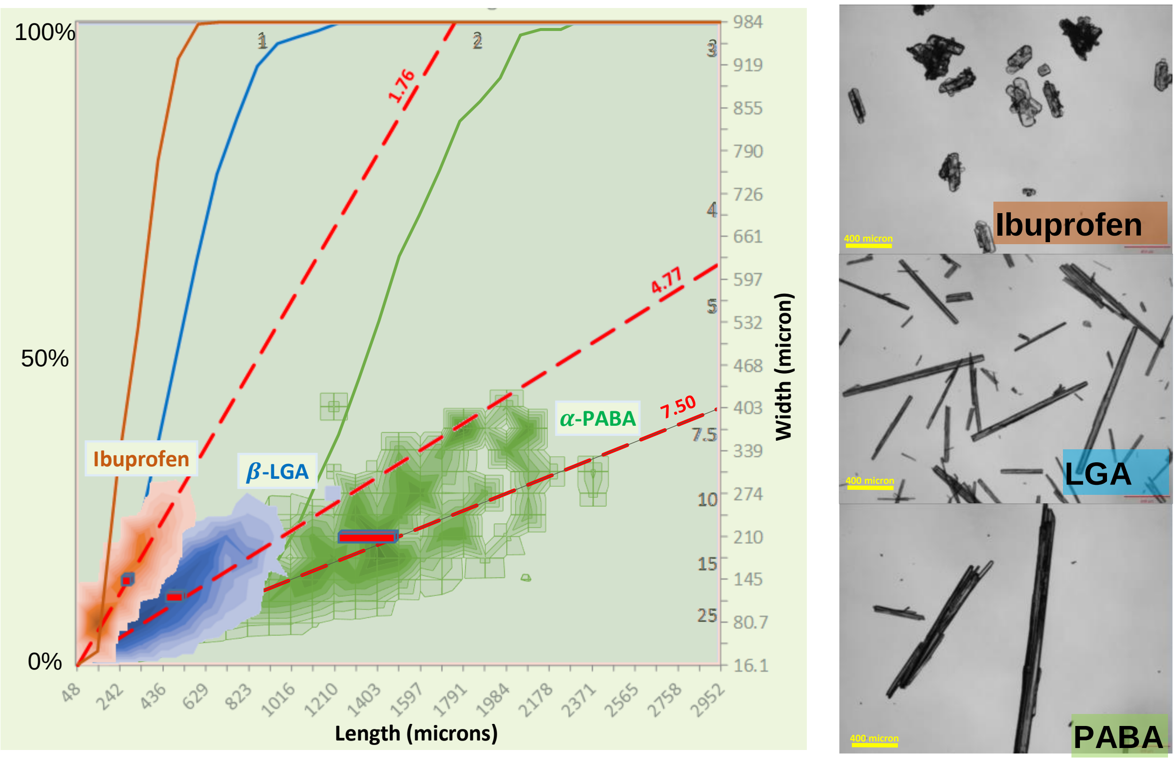

Figure 5: 2D volume based size distribution of the three reference materials (lightly filters crystallisation mass) used in this study: PABA ($L/W = 7.5$), LGA ($L/W = 4.8$), and Ibuprofen ($L/W = 1.8$). The dashed red lines represent the volume-averaged aspect ratio. Also depicted are the cumulative volume distribution of L (Eq. 20), solid lines, left axis)

6. Effect of pressure on the average particle length and width

Table 3: Distribution statistics for the **BOTTOM** samples.

**Bottom** Samples Materials	Inlet Pressure Bar	L_{ref} µm	W_{ref} µm	L_{av} µm	W_{av} µm	L_{av}/W_{av}	±StDev	L_{av}/L_{ref}	W_{av}/L_{ref}	<25% L_{max}	>90% L_{max}
	0.25*			464	108	4.3	0.5	1.00	0.23	307	758
	1.50			464	108	4.3	0.6	1.00	0.23	307	700
LGA	3.00	464	108	384	99	3.9	0.5	0.83	0.21	259	646
	4.00			351	103	3.4	0.4	0.76	0.22	226	630
	5.50			271	91	3.0	0.4	0.58	0.20	194	484
	0.25*			1355	181	7.5	0.4	1.00	0.13	1086	1947
	1.50			1207	200	6.0	0.4	0.89	0.15	979	1947
PABA	3.00	1355	200	1218	182	6.7	0.4	0.90	0.13	913	1874
	4.00			889	133	6.7	0.4	0.66	0.10	873	1851
	5.50			591	124	4.8	0.4	0.44	0.09	436	959
	0.25*			257	138	1.9	0.3	1.00	0.54	226	413
	1.50			210	128	1.6	0.3	0.82	0.50	145	379
Ibuprofen	3.00	257	138	158	92	1.7	0.2	0.61	0.36	113	306
	4.00			159	89	1.8	0.3	0.62	0.35	129	355
	5.50			155	89	1.7	0.3	0.60	0.35	118	316

* Reference sample, just vacuum filtered, not percolated

Table 4: Distribution statistics for the **TOP** samples.

**Top** Samples Materials	Inlet Pressure Bar	L_{ref} µm	W_{ref} µm	L_{av} µm	W_{av} µm	L_{av}/W_{av}	±StDev	L_{av}/L_{ref}	W_{av}/L_{ref}	<25% L_{max}	>90% L_{max}
	0.25*			464	108	4.3	0.5	1.00	0.23	307	758
	1.50			464	108	4.3	0.5	1.00	0.23	307	898
LGA	3.00	464	108	407	109	3.7	0.6	0.88	0.23	268	677
	4.00			462	107	4.3	0.5	1.00	0.23	275	744
	5.50			316	94	3.4	0.5	0.68	0.20	210	517
	0.25*			1355	200	6.8	0.4	1.00	0.15	1086	1947
	1.50			1297	203	6.4	0.4	0.96	0.15	908	1912
PABA	3.00	1355	200	1224	192	6.4	0.4	0.90	0.14	919	1791
	4.00			1201	183	6.6	0.5	0.89	0.14	580	1647
	5.50			1217	198	6.1	0.4	0.90	0.15	913	1887
	0.25*			257	138	1.9	0.3	1.00	0.54	226	413
	1.50			232	138	1.7	0.2	0.90	0.54	145	379
Ibuprofen	3.00	257	138	167	97	1.7	0.3	0.65	0.38	118	314
	4.00			195	100	2.0	0.3	0.76	0.39	113	306
	5.50			177	98	1.8	0.3	0.69	0.38	113	306

* Reference sample, just vacuum filtered, not percolated

7. 2D PSD as function of Pressure

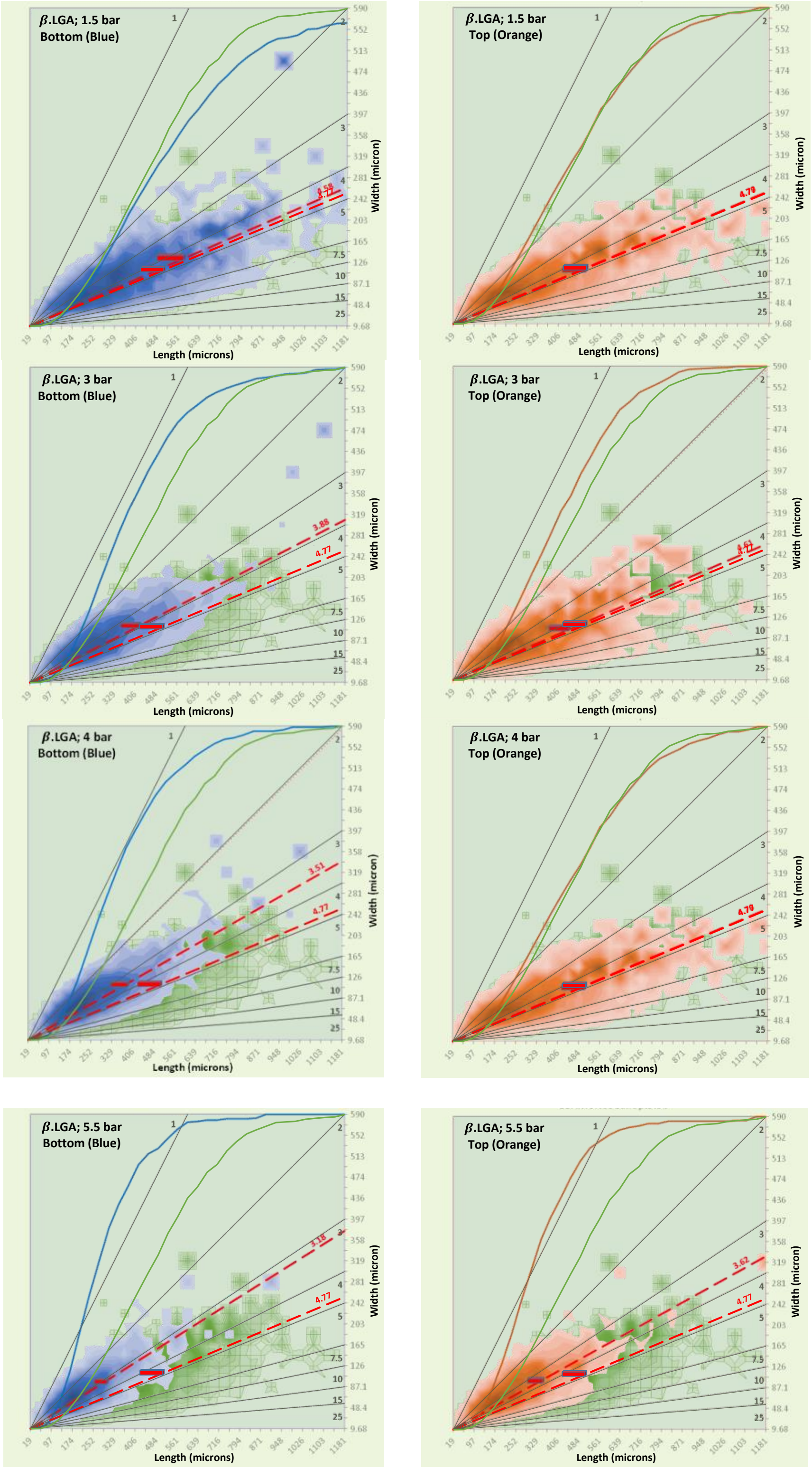

Bottom

W/L: no change

PSD: no change

Pattern: no change

Top

W/L: no change

PSD: no change

Pattern: no change

Bottom

W/L: : 4.8 → 3.9

PSD: F curve to lower L

Pattern: W constant

Top

W/L: no change

PSD: small reduction

Pattern: L,W both smaller

Bottom

W/L: 4.8 → 3.5

PSD: furhter F curve shift

Pattern: W constant

Top

W/L: no change

PSD: no change

Pattern: no change

Bottom

W/L: 4.8 → 3.2

PSD: furhter F curve shift

Pattern: W constant

Top

W/L: 4.8 → 3.6

PSD: F curve to lower L

Pattern: W constant

Particles snap (W constant), then fracture (L&W reduce)?

Figure 6: LGA results before (reference, green) and after (bottom of the bed in blue and top in orange) pressure percolation. Solid grey lines represent constant aspect ratio L/W (number given, ≥ 1 by definition). At different inlet pressure (ΔP_{inl})

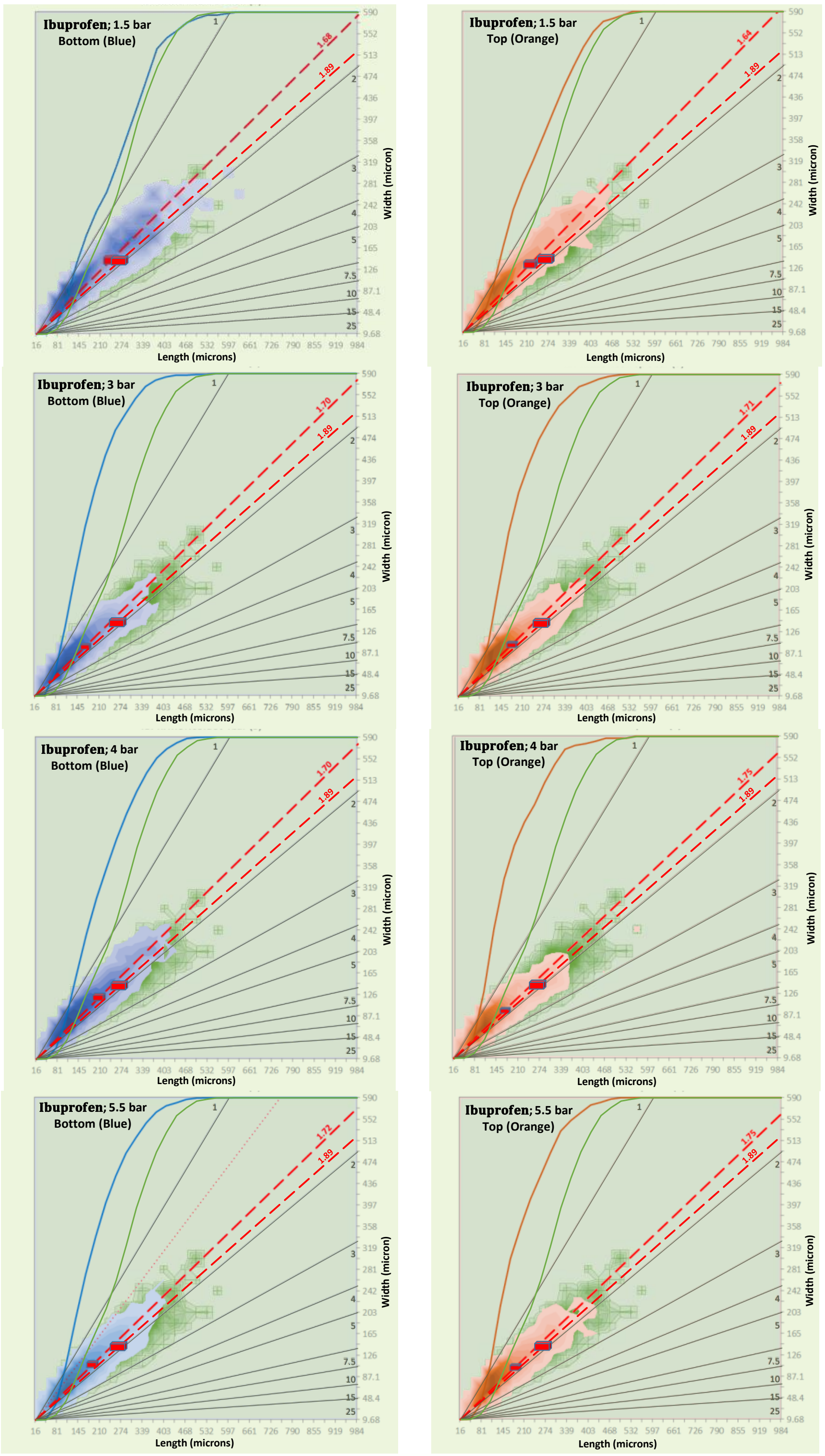

Bottom
W/L: 1.9 → 1.7
PSD: F curve shift
Pattern: W constant

Top
W/L: 1.9 → 1.6
PSD: F curve shift
Pattern: W,L reduce

Bottom
W/L: 1.9 → 1.7
PSD: F curve shift
Pattern: W/L constant

Top
W/L: 1.9 → 1.7
PSD: F curve shift more pronounced
Pattern: W/L constant

Bottom
W/L: 1.9 → 1.7
PSD: no further change
Pattern: W/L constant

Top
W/L: 1.9 → 1.7
PSD: no further change
Pattern: W/L constant

Bottom
W/L: 1.9 → 1.7
PSD: no further change
Pattern: W/L constant

Top
W/L: 1.9 → 1.7
PSD: no further change
Pattern: W/L constant

Figure 7: Ibuprofen results before (reference, green) and after (bottom of the bed in blue and top in orange) pressure percolation. Solid grey lines represent constant aspect ratio L/W (number given, ≥ 1 by definition). At different inlet pressure (ΔP_{inl})

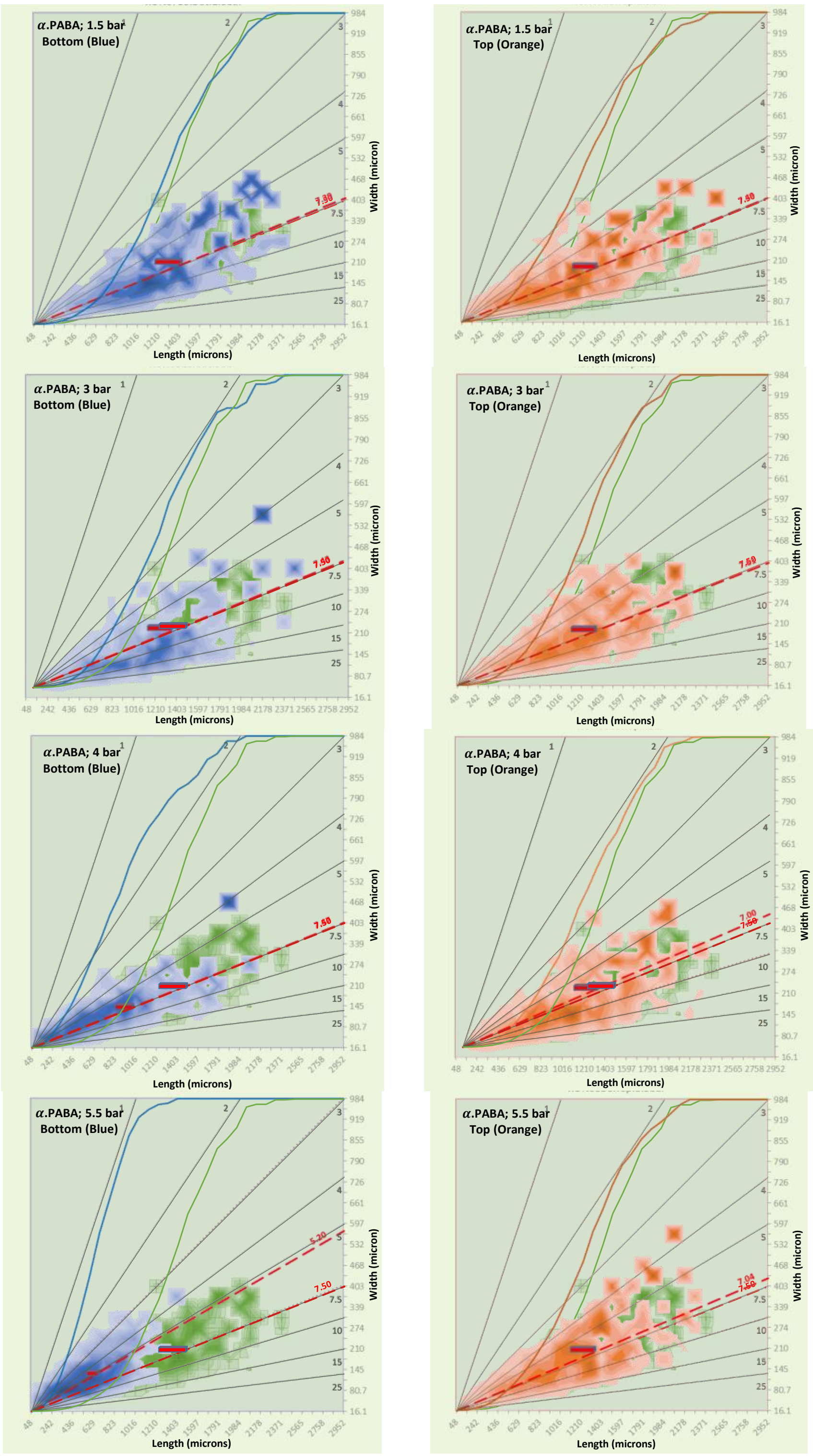

Bottom
W/L: no change
PSD: no change
Pattern: no change

Top
W/L: no change
PSD: no change
Pattern: no change

Bottom
W/L: no change
PSD: no change
Pattern: no change

Top
W/L: no change
PSD: no change
Pattern: no change

Bottom
W/L: 7.5 → 7.0
PSD: slight F curve shift
Pattern: L,W reduced

Top
W/L: no change
PSD: F curve to lower L
Pattern: L&W smaller

Bottom
W/L: 7.5 → 5.2
PSD: significant F curve shift
Pattern: L,W reduce

Top
W/L: no change
PSD: no change
Pattern: no change

Figure 8: PABA results before (reference, green) and after (bottom of the bed in blue and top in orange) pressure percolation. Solid grey lines represent constant aspect ratio L/W (number given, ≥ 1 by definition). At different inlet pressure (ΔP_{inl})

8. Effect of Scale

Figure 9 shows the 2D volume based size distribution for D-35 filter chamber (large-scale) pressure percolation of beta glutamic acid high aspect ratio crystals. In this work, the cakes were quite small after percolation and all samples were taken from bottom only.

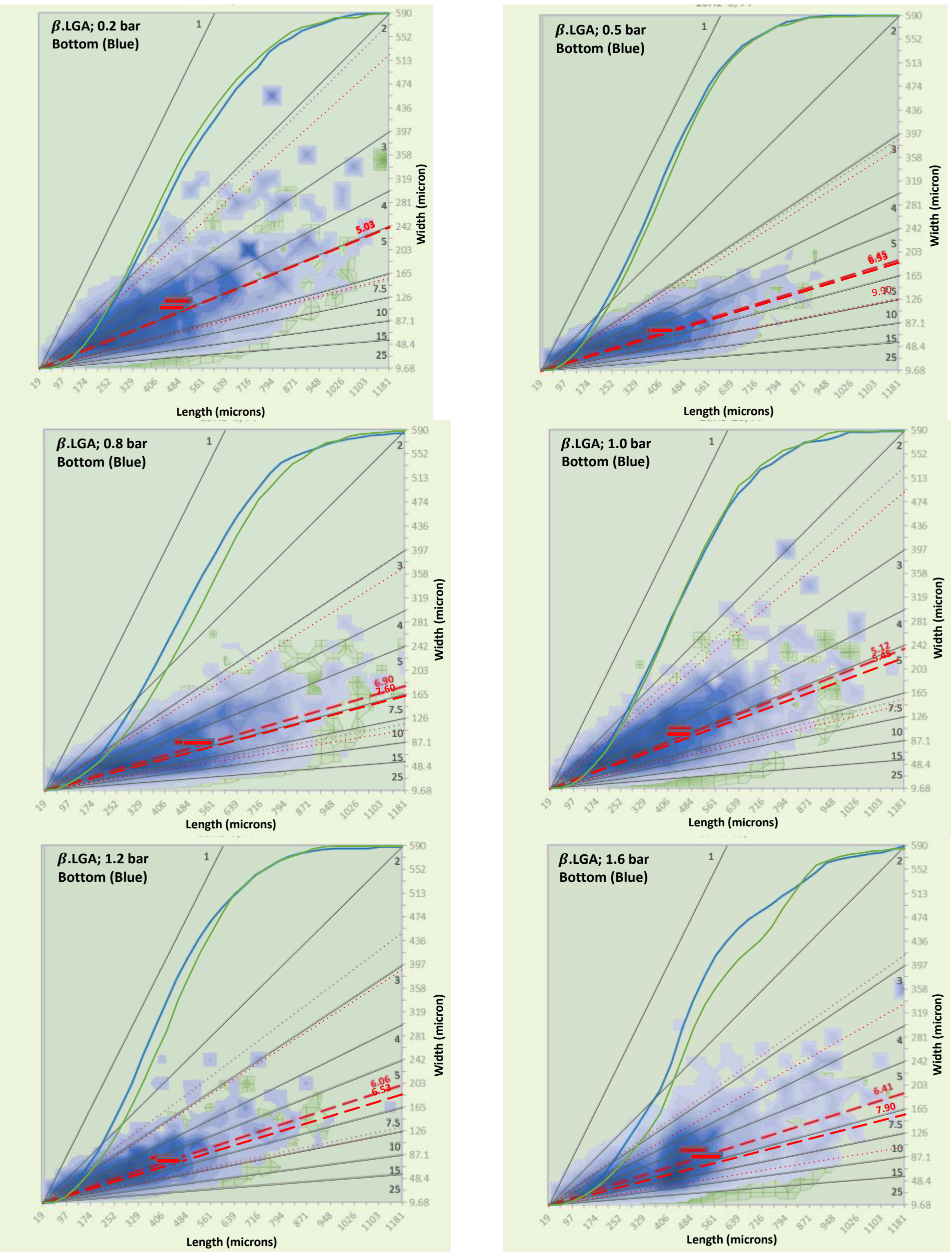

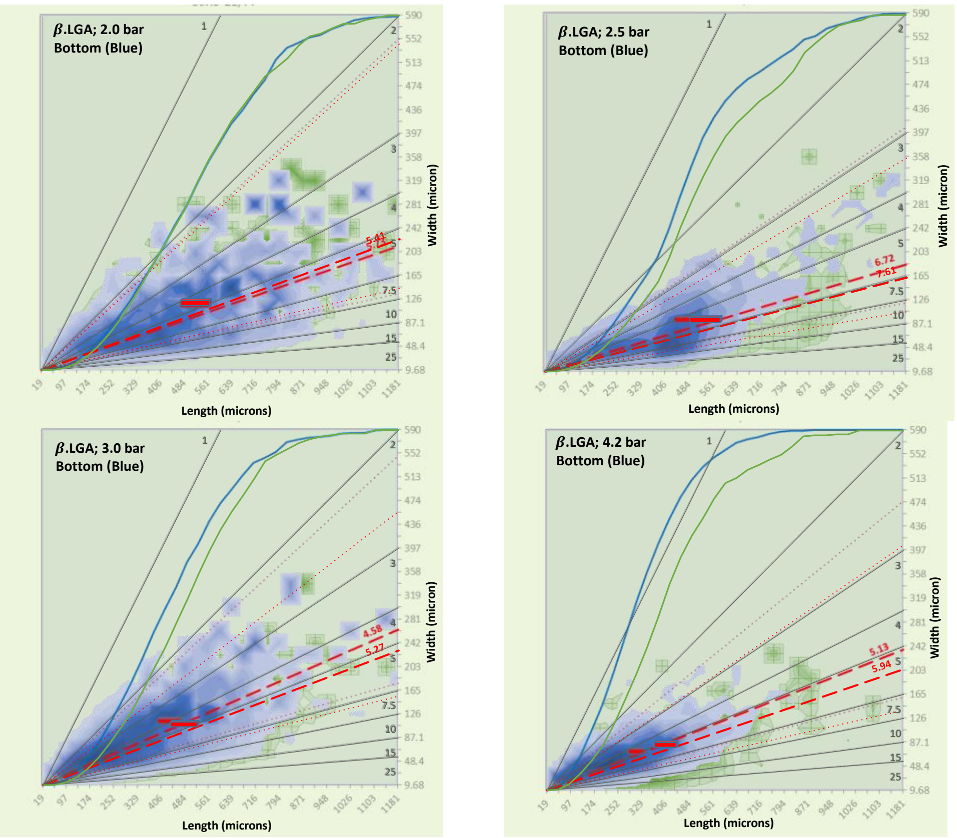

Figure 9: Data of large filter (ID-35) for beta glutamic acid at different inlet pressure (ΔP_{inl})

9. Enthalpy and entropy of sublimation

Table 5: All the physic properties references

Compound	Mw $g/mole$	Density g/L	$\Delta H_{S\to G}$ $kJ/mole$	$\Delta S_{S\to G}$ $kJ/mole$	Ref
**LGA**	147	1600	121	128	[1, 2]
**Ibuprofen**	207	1030	116	240	[3, 4]
**PABA**	137	1370	113	45	[5]
**Caffeine**	174	1230	105	269	[6]
**Theophylline**	180	1500	132	209	[6]
**Aspirin**	180	1400	119	235	[7]
**Paracetamol**	151	1260	118	190	[8]

Note: for caffeine and theophylline, the thermodynamic parameters where obtained by conversion of the vapour pressure correlation:

$$\ln\left(\frac{P}{Pa}\right) = \ln(10) \times \log(\frac{P}{Pa}) = \left(-\frac{A}{T} + B\right)\ln(10)$$

And

$$\Delta G_{S\to G} = -RTLn\left(\frac{P}{Po}\right) = R \times A\ln(10) + R\,T(\ln(Po) - B\ln(10)),$$

$$with\ Po = 0.1013\ MPa$$

It follows that

$$\Delta H_{S\to G} = R \times A \times \ln(10)$$

And

$$\Delta S_{S\to G} = \Delta G_{S\to G} - \Delta H_{S\to G} = R \times (\ln(Po) - B\ln(10))$$

The volumetric Gibbs free energy is calculated as:

$$\Delta G_{S\to G} = (\Delta H_{S\to G} + 293 \times \Delta S_{S\to G}) \times \frac{\rho}{Mw}$$

With ρ the solid density in g/L and Mw the molecular weight in $g/mole$

Reference

[1] A.M. Booth, M.H. Barley, D.O. Topping, G. McFiggans, A. Garforth, C.J. Percival, Solid state and sub-cooled liquid vapour pressures of substituted dicarboxylic acids using Knudsen Effusion Mass Spectrometry (KEMS) and Differential Scanning Calorimetry, Atmos. Chem. Phys. 10(10) (2010) 4879-4892.

[2] J.S. Gaffney, R.C. Pierce, L.J.J.o.t.A.C.S. Friedman, Mass spectrometer study of evaporation of. alpha.-amino acids, 99(13) (1977) 4293-4298.

[3] R. Maxwell, J. Chickos, An Examination of the Thermodynamics of Fusion, Vaporization, and Sublimation of Ibuprofen and Naproxen by Correlation Gas Chromatography, Journal of Pharmaceutical Sciences 101(2) (2012) 805-814.

[4] G.L. Perlovich, S.V. Kurkov, L.K. Hansen, A. Bauer-Brandl, Thermodynamics of sublimation, crystal lattice energies, and crystal structures of racemates and enantiomers: (+)- and (+/-)-ibuprofen, J Pharm Sci 93(3) (2004) 654-66.

[5] C.G. de Kruif, J. Voogd, J.C.A. Offringa, Enthalpies of sublimation and vapour pressures of 14 amino acids and peptides, The Journal of Chemical Thermodynamics 11(7) (1979) 651-656.

[6] U.J. Griesser, M. Szelagiewicz, U.C. Hofmeier, C. Pitt, S. Cianferani, Vapor Pressure and Heat of Sublimation of Crystal Polymorphs, Journal of Thermal Analysis and Calorimetry 57(1) (1999) 45-60.

[7] G.L. Perlovich, S.V. Kurkov, A.N. Kinchin, A. Bauer-Brandl, Solvation and hydration characteristics of ibuprofen and acetylsalicylic acid, AAPS PharmSci 6(1) (2004) 22-30.

[8] G.L. Perlovich, T.V. Volkova, A. Bauer-Brandl, Polymorphism of paracetamol, Journal of Thermal Analysis and Calorimetry 89(3) (2007) 767-774.
